# Bioactive Chitosan Oligosaccharides Derived from Crab Shell Waste Stimulate Osteogenic Differentiation in Pre-Osteoblast Cells

**DOI:** 10.3390/md24090302

**Published:** 2026-08-30

**Authors:** Supawadee Duangprom, Piyapon Janpan, Sineenart Songkoomkrong, Siriporn Namwongsa, Prateep Amonruttanapun, Jirawat Saetan, Yujun Sung, Vichapol Prasattongosoth, Yutthakan Saengkun, Montakan Tamtin, Prasert Sobhon, Napamanee Kornthong

**Affiliations:** 1 Chulabhorn International College of Medicine, Thammasat University, Rangsit Campus, Pathumthani 12120, Thailand; su.duangprom@gmail.com (S.D.); piyapon.ater@gmail.com (P.J.); sineenartsong@gmail.com (S.S.); siriphorn.nonkhaow@gmail.com (S.N.); wanderer_sci@yahoo.com (P.A.); abuxas.saengkun@gmail.com (Y.S.); 2 Research Unit in Innovative Marine Biotechnology and Natural Bio-Resources for Sustainable Health and Wellness, Thammasat University, Pathumthani 12120, Thailand; ethansung416@gmail.com (Y.S.); roccor123r@gmail.com (V.P.); 3 Division of Health and Applied Sciences, Faculty of Science, Prince of Songkla University, Hat Yai, Songkhla 90110, Thailand; jisaetan@gmail.com; 4 Department of Fisheries, Ministry of Agriculture and Cooperatives, Lat Yao, Chatuchak, Bangkok 10900, Thailand; montakan.t14@gmail.com; 5 Department of Anatomy, Faculty of Science, Mahidol University, Bangkok 10400, Thailand; prasert.sob@mahidol.ac.th

**Keywords:** chitosan, chitosan oligosaccharide (COS), transcriptomics, osteogenesis

## Abstract

Crustacean shell waste from the seafood industry represents an underutilized food waste with the potential to produce chitosan and chitosan oligosaccharides (COS), with promising applications in bone regeneration. However, the molecular mechanisms of COS during osteogenic differentiation remain uncharacterized. In this study, chitosan was extracted from mud crab (*Scylla olivacea*) shell waste and COS was prepared from chitosan by acid hydrolysis and then characterized by FT-IR and FESEM, confirming the chitosan backbone and a degree of deacetylation (DD) exceeding 80%. The COS treatment in the concentration range of 5–320 µg/mL significantly enhanced MC3T3-E1 pre-osteoblast proliferation without cytotoxicity, and it increased alkaline phosphatase (ALP) activity, extracellular matrix mineralization, and Runx2/osterix (OSX) expression. Transcriptomics of pre-osteoblasts treated with COS at 320 µg/mL for 21 days identified 19,643 differentially expressed genes, revealing coordinated activation of multiple osteogenesis-related signaling pathways, including ECM–integrin/focal adhesion, PI3K-Akt/MAPK, TGF-β/BMP, Wnt/β-catenin, and parathyroid hormone (PTH) synthesis. These findings demonstrate that marine-derived COS promotes osteoblast differentiation through the coordinated activation of multiple signaling pathways, providing the first comprehensive transcriptomic characterization of MC3T3-E1 pre-osteoblast directly treated with COS during osteogenic differentiation. Therefore, this study supports the use of COS as a promising bioactive oligosaccharide for bone regeneration and osteoporosis management, and further in vivo and clinical studies are recommended.

## 1. Introduction

The global seafood processing industry generates enormous quantities of shell waste annually, presenting both an environmental burden and an untapped biological resource [1]. The seafood industry uses approximately 25–30% of the total biomass for human consumption [2], while the remaining fractions are discarded as waste; consequently, the aquaculture and seafood-processing industries generate a substantial amount of waste on a global scale each year [3]. Crustacean shells—primarily from crab, shrimp, and lobster—constitute approximately 25–30% of total aquaculture biomass discarded as waste, with nearly 8 million tons of crab, shrimp, and lobster shells produced globally each year [4]. These shells are rich in chitin (15–40%), protein (20–40%), and calcium carbonate (20–50%), making them highly valuable raw materials for biomaterial extraction [5]. The accumulation of such seafood processing waste poses significant environmental challenges, particularly in low-income countries with inadequate waste management infrastructure, underscoring the urgency of developing sustainable valorization strategies within a circular bioeconomy framework [6].

Chitin, the second most abundant natural polysaccharide after cellulose, is the principal structural component of crustacean exoskeletons and is not exclusive to marine invertebrates; it is also found in the cell walls of fungi, cuticles of insects, and squid pens [7,8]. Chitosan, derived from chitin through deacetylation, is a cationic polysaccharide composed of β-1,4-linked glucosamine and N-acetyl-glucosamine units (C_6_H_11_NO_4_)_n_, whose degree of deacetylation (DD) is a critical physicochemical parameter that directly determines solubility, charge density, biological activity, and downstream processing performance [7,8,9]. The DD is widely used to classify chitosan quality. Values of 55–70% are categorized as low, 70–85% as medium, 85–95% as high, and 95–100% as ultrahigh deacetylation, with higher DD generally associated with enhanced solubility, electrostatic interaction with cellular membranes, and overall biological activity [10,11]. Chitosan oligosaccharides (COSs) are low-molecular-weight derivatives of chitosan, typically ranging from 1 to 10 kDa with a degree of polymerization (DP) of 2 to 20 saccharide units, produced through controlled chemical or enzymatic hydrolysis of the chitosan polymer chain [12,13]. This reduced molecular size confers COSs with complete water solubility at physiological pH, superior bioavailability, enhanced biodegradability, and negligible cytotoxicity compared to native chitosan, rendering COSs highly suitable for a wide range of biomedical applications including drug delivery, antioxidant therapy, anti-inflammatory treatment, and bone regeneration [11,12,14].

The molecular characterization of COS derived from crustacean shell waste has been extensively reported using FT-IR spectroscopy, confirming that the core polysaccharide backbone structure is preserved following controlled depolymerization [7,15,16]. These spectral profiles have been consistently reproduced across COSs derived from diverse crustacean sources including crab, shrimp, and lobster shells, confirming that low-molecular-weight COS retains the molecular identity of native chitosan while exhibiting the improved solubility and bioavailability characteristic of chain-shortened derivatives [15,17]. The cationic nature of COSs, arising from protonated free amino groups (–NH_3_^+^) at physiological pH, enables electrostatic interaction with the negatively charged osteoblast plasma membrane—facilitating membrane binding and cellular uptake through endocytic pathways including clathrin-mediated endocytosis and macropinocytosis [18,19,20]. Building on this signaling framework, COSs have shown direct osteogenic activity across multiple experimental models relevant to osteoporosis, which is a metabolic bone disease arising from an imbalance between osteoblast-mediated bone formation and osteoclast-mediated resorption [21]. Hou et al. [22] reported that water-soluble chitosan significantly increased ALP activity, cell viability, and mineralization while reducing ROS levels in MC3T3-E1 pre-osteoblasts. Osteoblasts plays an important role in maintaining bone homeostasis, and promoting osteoblast differentiation is a feasible method to prevent osteoporosis [23]. Chitosan has been reported to modulate key intracellular signaling pathways in bone-forming cells, including the PI3K/Akt and MAPK pathways that regulate cell survival, proliferation, and differentiation [18,19]. The TGF-β/BMP signaling axis operating through receptor-Smad mediators including Smad1, Smad4, Smad5, and Smad9 is well recognized as a master regulator of osteoblast commitment and bone matrix formation [24]. Wnt signaling, through stabilization of β-catenin, directly activates Runx2 gene expression and promotes osteoblast differentiation and bone formation [21,25]. The convergence of PI3K/Akt, MAPK, TGF-β/BMP, and Wnt pathways on the master transcription factors Runx2 and osterix (OSX) drives ALP activity, collagen synthesis, and extracellular matrix mineralization that define functional osteoblast maturation [24,26,27]. Most recently, Vimalraj et al. [28] demonstrated that COS promoted Runt-related transcription factor 2 (Runx2), collagen, osteocalcin (OCN), and osteonectin (ON) expression and reduced tartrate-resistant acid phosphatase (TRAP) activity—a marker of osteoclast activity—in mouse mesenchymal stem cell (MSC) cultures, and attenuated dexamethasone-induced bone loss with improved mineralization in a zebrafish osteoporosis model.

While COS have demonstrated osteogenic effects across multiple cell models, the molecular mechanisms underlying these effects remain incompletely characterized. Existing studies have predominantly employed targeted single-gene or single-protein approaches—such as RT-qPCR for Runx2 and ALP, or Western blot for individual osteogenic markers—without comprehensively mapping the simultaneous activation of multiple signaling networks [19,28]. Furthermore, transcriptome-wide gene expression profiling of COS effects in pre-osteoblast cells has not been reported; the only gene expression study to date employed a limited cDNA microarray panel identifying only 16 differentially expressed genes, leaving the global transcriptomic basis of COS osteogenic action uncharacterized [19]. Mechanistic evidence directly linking COS derived from marine crustacean shell waste to comprehensive osteoblast signaling network remodeling therefore remains absent from previous studies. The present study therefore aimed to (1) extract and characterize chitosan from crab shell waste; (2) prepare water-soluble COS via controlled oxidative depolymerization; (3) evaluate the effects of COS on MC3T3-E1 pre-osteoblast viability, ALP activity, mineralization, and osteogenic gene and protein expression; and (4) investigate the molecular mechanisms underlying COS-induced osteogenic differentiation through transcriptome profiling and signaling pathway analysis.

## 2. Results

### 2.1. Characterization of Chitosan Samples

All chitosan samples, including crab shell chitosan and chitosan oligosaccharide (COS), were obtained as white powders. FT-IR analysis was further performed to identify the functional groups of the chitosan samples in comparison with standard chitosan and COS. The FT-IR spectrum and degree of deacetylation (DD) are presented in Table 1 and Supplementary Table S1.

**Figure 1:**
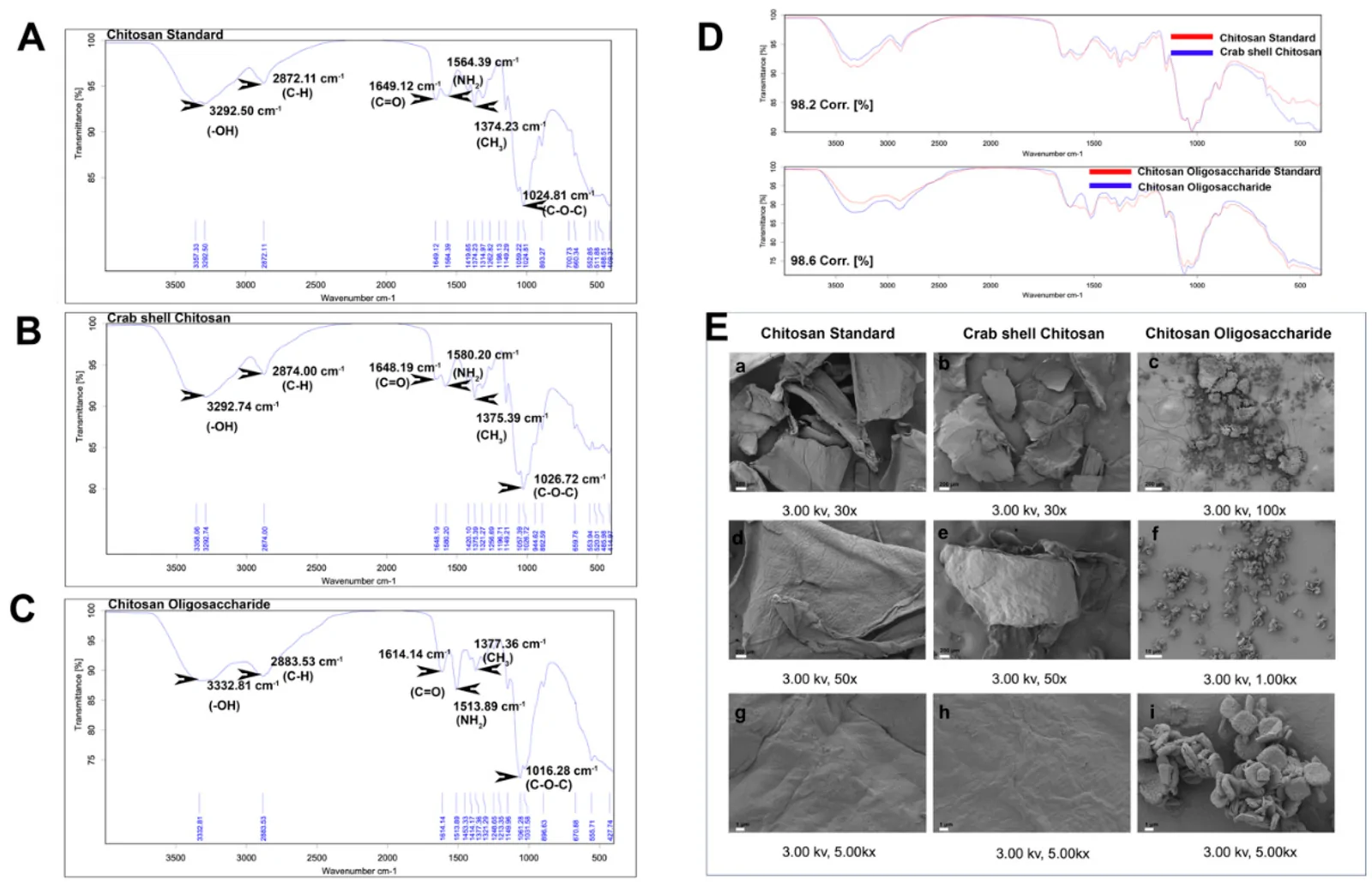
FT-IR spectra and morphological characterization of chitosan samples prepared from crab shells, compared with a chitosan standard. (**A**) FT-IR spectrum of the standard chitosan. (**B**) FT-IR spectrum of chitosan prepared from crab shells. (**C**) FT-IR spectrum of chitosan oligosaccharide (COS) derived from acid-hydrolyzed chitosan. (**D**) Comparison of FT-IR spectra of crab shell chitosan and standard chitosan and chitosan oligosaccharide and standard chitosan. (**E**) Field emission scanning electron microscopy (FESEM) images showing the surface morphology of the chitosan samples (a, d, g representative standard chitosan in each magnifications (30×–5000×), b, e, g representative Crab shell chitosan in each magnifications (30×–5000×), c, f, i representative chitosan oligosaccharide in each magnifications (30×–5000×).

Five prominent peaks were identified in the FT-IR spectrum of standard chitosan, which represent its characteristic functional groups (Figure 1A). These bands consisted of an absorption peak at 3292.50 cm^−1^ corresponding to hydroxyl (–OH) stretching vibrations, at wavenumber 2872.11 cm^−1^ indicating stretching vibrations of aliphatic C–H groups (–CH and –CH_2_), at wavenumber 1649.12 cm^−1^ representing the amide I band, specifically the C=O stretching vibration of the acetylated units, a band peak at 1564.39 cm^−1^ attributed to N–H bending vibrations of primary amine (–NH_2_) groups (amide II), and a band peak at 1024.81 cm^−1^ indicating C–O–C stretching and C–O stretching in the saccharide ring. The FT-IR spectrum of standard chitosan is consistent with previous studies by Kannan et al. [29] and Karthickeyan et al. [16].

The FT-IR spectrum in Figure 1B shows characteristic bands of the acid-hydrolyzed chitosan derived from crab shell at wavenumbers 3292.74, 2874.00, 1648.19, 1580.20, 1420.10, and 1026.72 cm^−1^. These peaks correspond to O–H stretching vibrations, aliphatic C–H (–CH, –CH_2_) stretching vibrations, amide I (C=O stretching), amide II (N–H bending of –NH_2_), methyl group (–CH_3_) vibrations, and C–O–C stretching of the ether bridge associated with β-(1→4)-glycosidic linkages between sugar subunits, respectively [7,16]. In addition, a band at 892.59 cm^−1^ is attributed to C–H deformation associated with the β-glycosidic linkage [7]. The degree of deacetylation of crab shell chitosan was determined to be 85.78% and 81.65% by FT-IR and titration methods (Supplementary Table S1). The FT-IR spectrum of COS prepared by acid hydrolysis exhibited a high similarity to the characteristic absorption bands of the standard chitosan, with a spectral matching score of 98.2% (Figure 1C). This indicates that the chitosan backbone was retained after hydrolysis and demonstrates the successful production of COS from crab shell-derived chitosan.

Field emission scanning electron microscopy (FESEM) was utilized to characterize the surface morphology, porosity, and particle size of chitosan samples at varying magnifications (30×–5000×) (Figure 1E). The FESEM images demonstrated structural differences among the chitosan samples. FESEM imaging revealed that crab shell-derived chitosan exhibited a dense, lamellar morphology with a relatively compact structural organization. Although the particle size remained comparatively large, this chitosan material showed a higher degree of fragmentation than the standard chitosan. At higher magnification, surface depressions and small pores were observed. In contrast, the chitosan oligosaccharide (COS), a low-molecular-weight chitosan, demonstrated a different microstructure and surface morphologies compared with the standard chitosan and crab shell chitosan, consisting of aggregated fine particles with a porous, sponge-like architecture. At high magnifications, the prepared COS revealed a crosslinking of pores and cavities. The particles were significantly smaller and had an irregular surface, consistent with extensive depolymerization.

### 2.2. Effect of Chitosan Oligosaccharide (COS) on MC3T3-E1 Cell Viability

**Figure 2:**
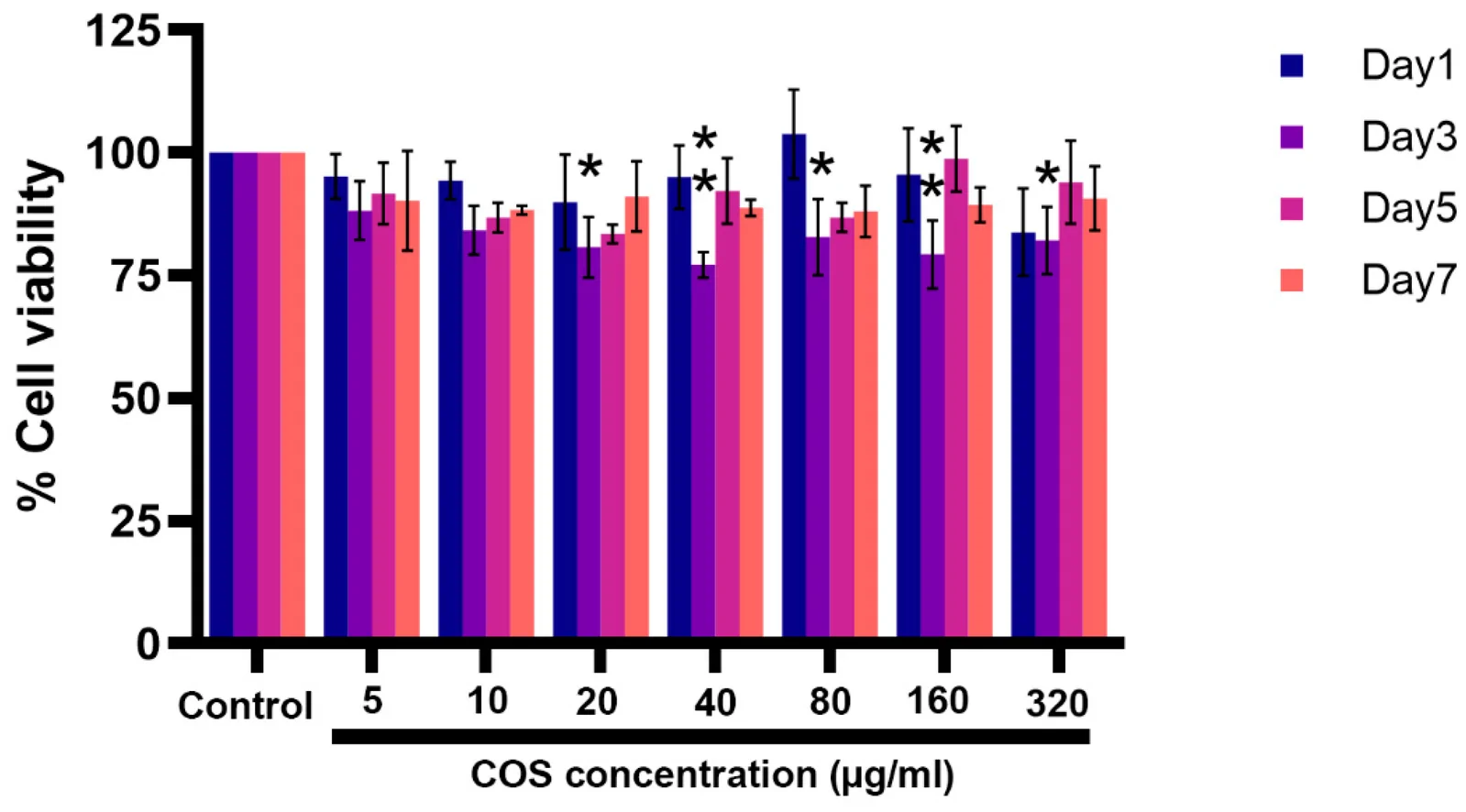
Evaluation of cell viability proportion by the MTT assay. The cytotoxic effect of COS extract on the proliferation of MC3T3-E1 cells on day 1, day 3, day 5, and day 7. Pre-osteoblast cells were treated with different concentrations (5–320 µg/mL) of COS extract. Cell viability of COS treated groups is shown as the percentage compared to the control group on each day. COS affected the cell viability of MC3T3-E1 cells. Data are expressed as a percentage compared with the control and shown as mean ± SEM (*n* = 3). The statistically significant differences were defined using one-way ANOVA at (*) *p* < 0.05, (**) *p* < 0.01.

To investigate the effect of COS on the viability MC3T3-E1 cells, various concentrations (5–320 µg/mL) of COS were added to the culture media on days 1, 3, 5, and 7. Compared to the control, the COS showed stimulatory effects on pre-osteoblast cell proliferation at all tested concentrations from 5 to 320 µg/mL (Figure 2). No cytotoxic effects were observed on the osteoblastic MC3T3-E1 cells. We further examined the effects of COS of various concentrations on enhancing MC3T3-E1 cells’ differentiation.

### 2.3. Effect of Chitosan Oligosaccharide on MC3T3-E1 Cell Functions

**Figure 3:**
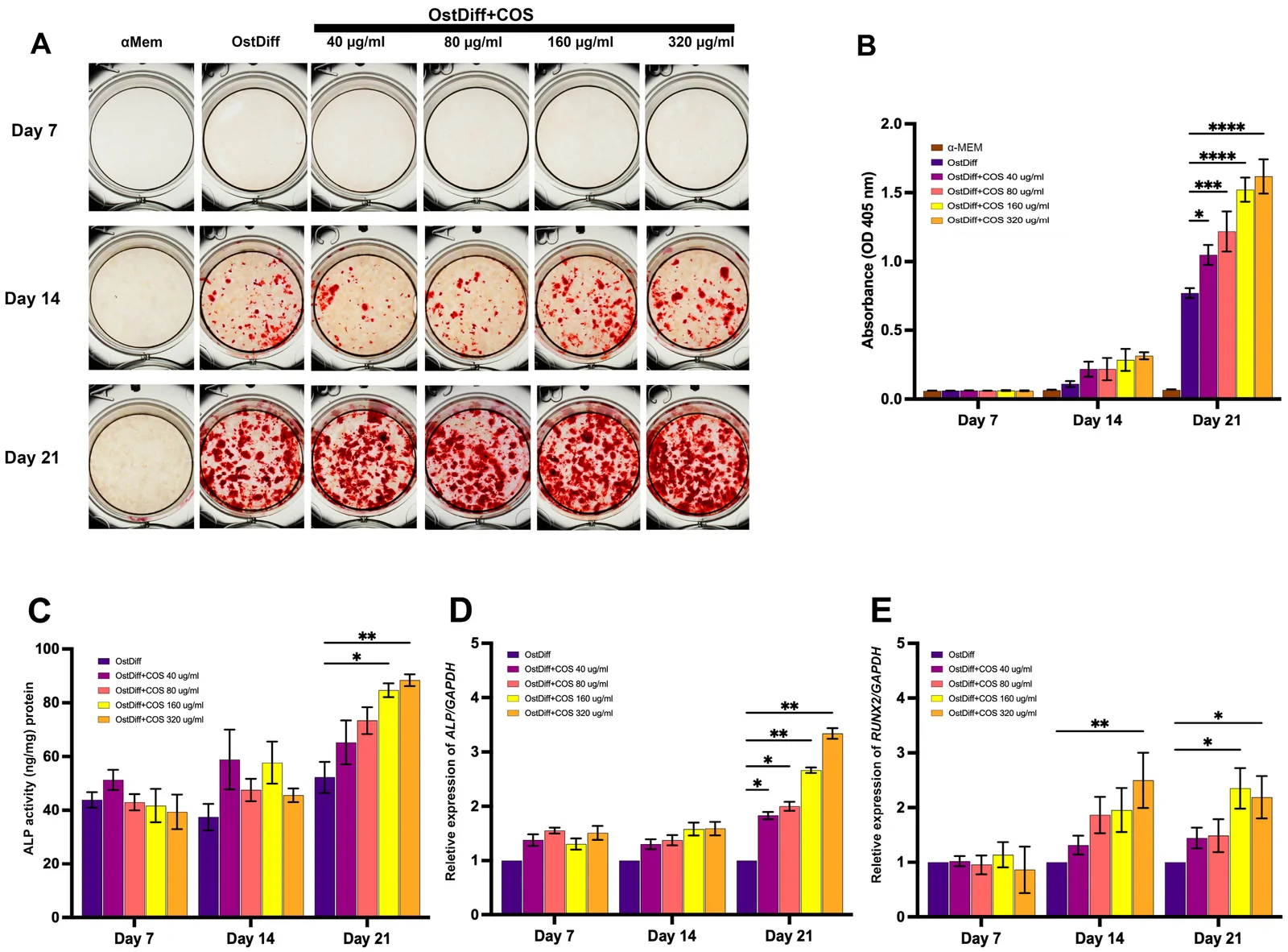
(**A**) Positive Alizarin Red S staining of COS-treated MC3T3-E1 cells in osteogenic differentiation medium compared to MC3T3-E1 cells cultured in osteogenic differentiation medium (OstDiff) without COS for 21 days. (**B**) Calcium deposition in the extracellular matrix was quantified following staining of mineralized calcium nodules with Alizarin Red S (ARS). (**C**) Alkaline phosphatase (ALP) activity assay. (**D**,**E**) Gene expression of *ALP* and *Runx2* was measured by RT-qPCR after 7, 14, and 21 days of treatment with COS (40, 80, 160, and 320 µg/mL) on MC3T3-E1 cells. All data represent mean ± SEM of five independent experiments. (*) *p* < 0.05, (**) *p* < 0.01, (***) *p* < 0.005, (****) *p* < 0.0001, versus induced group. ARS: Alizarin Red S staining, ALP: alkaline phosphatase, *Runx2*: RUNX family transcription factor 2, OstDiff: osteogenic differentiation medium, COS: chitosan oligosaccharide.

To investigate the effect of COS on osteogenic differentiation of MC3T3-E1 cells, alkaline phosphatase (ALP) activity was evaluated as an early marker of osteoblast differentiation (Figure 3C). Treatment with COS (40, 80, 160, and 320 µg/mL) significantly increased ALP activity compared with the untreated control (α-MEM), indicating enhanced early-stage osteogenic differentiation. In the ALP assay, alkaline phosphatase catalyzed the hydrolysis of p-nitrophenyl phosphate to produce the yellow-colored product p-nitrophenol, which was quantified spectrophotometrically. The ALP activity exhibited similar expression patterns in cells cultured on COS substrates, as shown in Figure 3C. Extracellular matrix mineralization is a hallmark of late-stage osteoblast differentiation and plays a critical role maintaining bone tissue mechanical integrity; mineralization was therefore assessed. As shown in Figure 3A, the increase in mineralization was significant in MC3T3-E1 cells in the presence of COS. Formation of bone involves a complex series of events that includes proliferation and differentiation of osteoprogenitor cells, which eventually result in the formation of a mineralized extracellular matrix. Thus, COS may have had direct stimulation effects on bone formation in the cultured MC3T3-E1 osteoblast cells.

Changes in the mRNA expression of the osteogenic differentiation-related genes in MC3T3-E1 cells after induction were determined at 7, 14, and 21 days. There was no significant difference in expression of *ALP* and *Runx2* between the groups at 7 days, and the expression of Runx2 was significantly higher at 21 days than at 14 days (*p* < 0.05) (Figure 3E). After 21 days, *ALP* expression was significantly increased in the COS 320 µg/mL group compared with control (*p* < 0.05) (Figure 3D).

**Figure 4:**
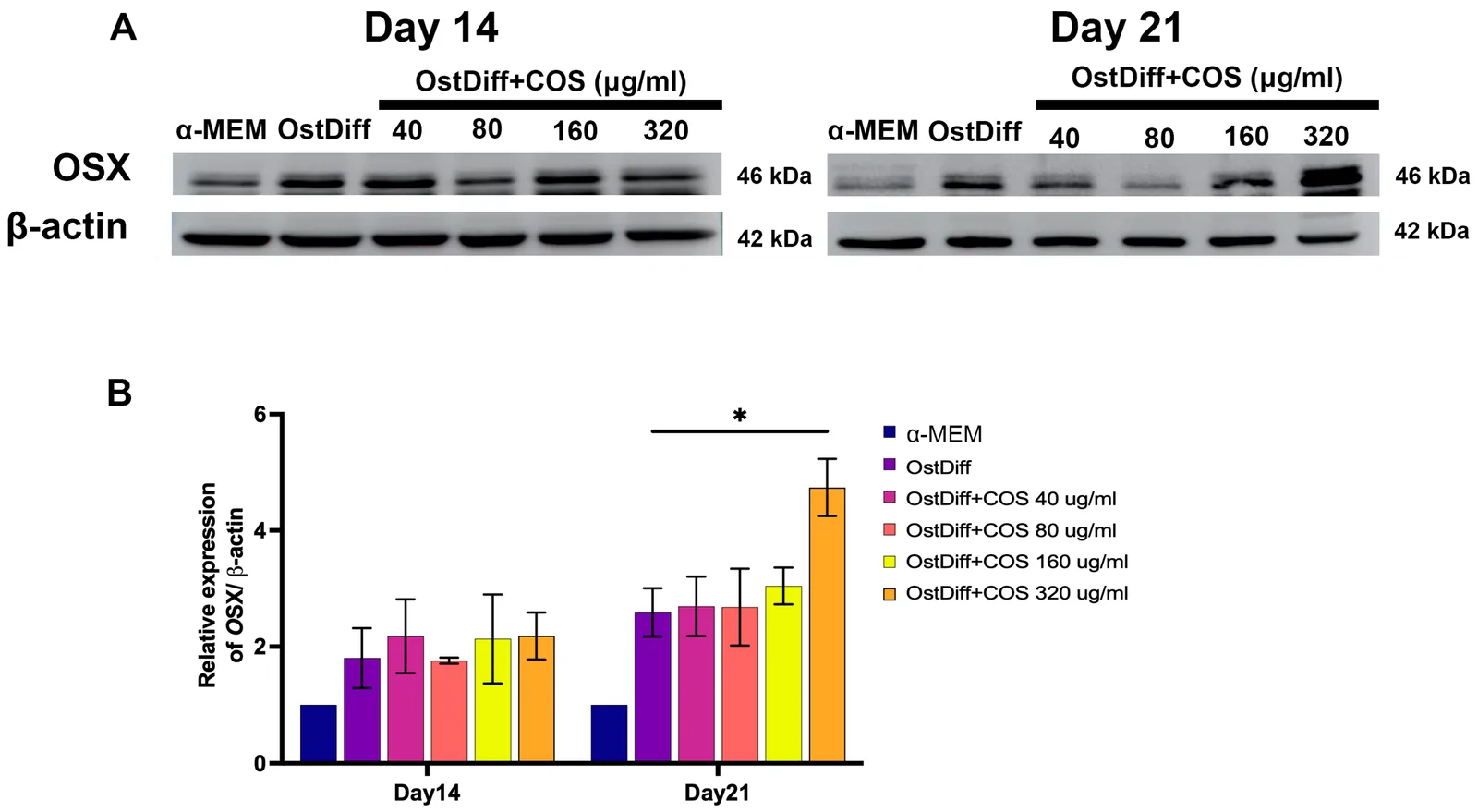
(**A**). Protein levels of osteogenesis differentiation marker osterix (OSX) induced by COS on day 14 and day 21. (**B**). Graphs show the levels of OSX protein on COS-treated (40–320 µg/mL) MC3T3-E1 cells in osteogenic differentiation medium compared to MC3T3-E1 cells cultured in osteogenic differentiation medium (OstDiff) without COS as control for 14 and 21 days. All data represent mean ± SEM of three independent experiments (*n* = 3). (*) *p* < 0.05.

The changes in expression of osteogenic differentiation related proteins in MC3T3-E1 cells were evaluated after 14 and 21 days of treatment with different concentrations of COS. As shown in Figure 4, OSX, a marker of late osteogenic differentiation, in each group at 21 days was higher than that at 14 days. In addition, OSX protein expression was significantly higher on MC3T3-E1 cells treated with COS at 320 µg/mL compared with the control (*p* < 0.05) (Figure 4 and Supplementary Figure S1).

### 2.4. Transcriptome Analysis

**Figure 5:**
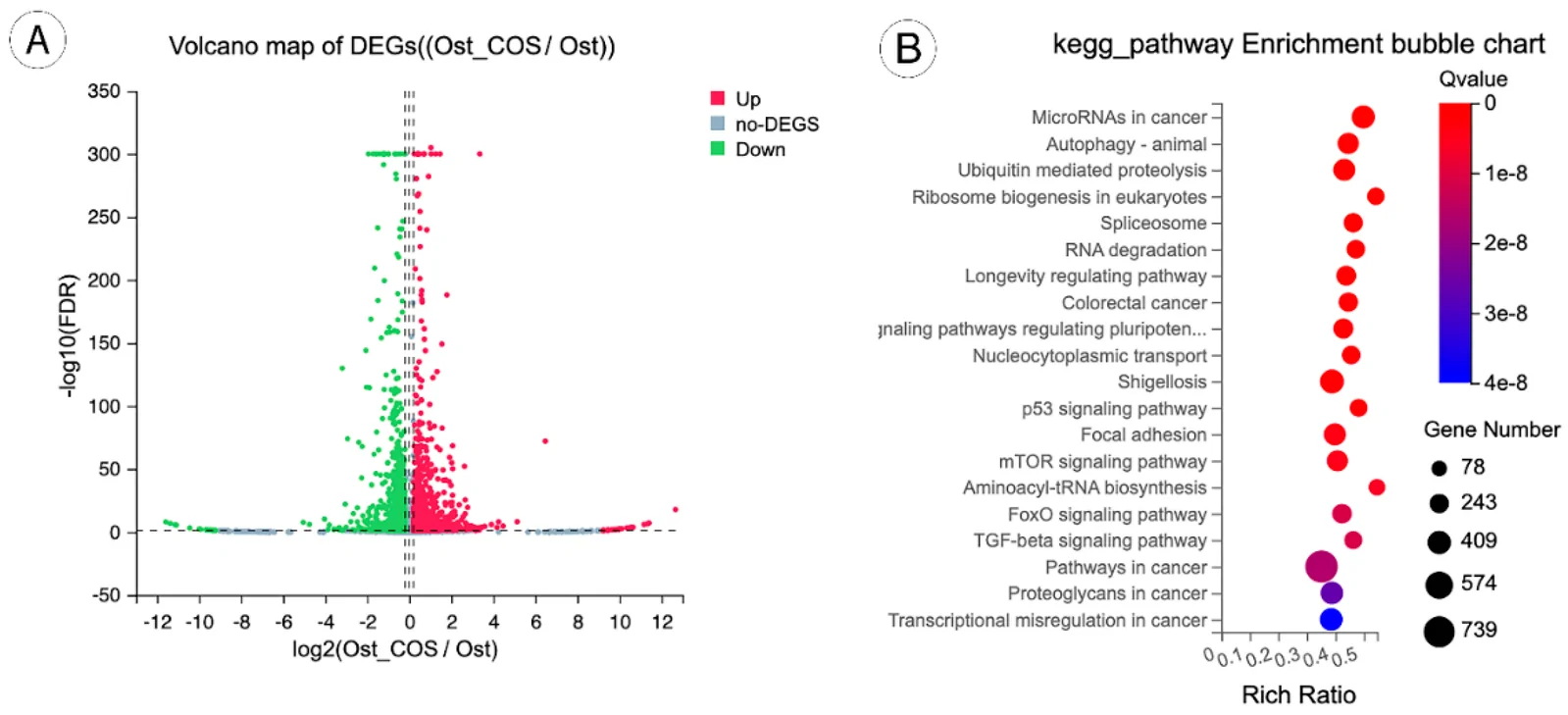
Identification and functional analysis of differentially expressed genes (DEGs) in osteogenic differentiation control (Ost) compared with COS-treated MC3T3-E1 preosteoblast (Ost_COS). (**A**) Volcano plot of DEGs analyzed by DESeq2 (log_2_ fold change ≥ 0.2; adjusted *p*-value [Qvalue] ≤ 0.05); red dots represent significantly upregulated DEGs, green dots represent significantly downregulated DEGs, and gray dots represent genes that did not exhibit statistically significant differential expression. (**B**) KEGG pathway enrichment analysis of differentially expressed genes (DEGs) in COS-treated MC3T3-E1 preosteoblast (Ost_COS) compared with the osteogenic differentiation control (Ost). Bubble chart displaying the top enriched KEGG pathways; bubble size represents number of DEGs enriched in each pathway and bubble color indicates adjusted *p*-value (Q value).

Transcriptome analysis of MC3T3-E1 pre-osteoblast treated with osteogenic induction medium (control) and COS at 320 μg/mL was compared. Each transcriptome generated an average of 6.65 gigabytes of raw read, with a total of 22,833 genes detected across all conditions. The average genome mapping ratio was 98.06%, while the average gene alignment rate was 84.15%. Pearson correlation coefficients between biological replicates exceeded 0.970 (Supplementary Figure S2), confirming high reproducibility. Gene expression distributions and density profiles were consistent across samples (Supplementary Figures S3 and S4). Comparison between two groups could identify 19,643 DEGs. Of these, 9237 were upregulated and 10,406 were downregulated (Figure 5A, Supplementary Table S2).

### 2.5. Gene Ontology (GO) Enrichment Analysis

The most enriched Gene Ontology (GO) terms were identified across cellular components (CCs), molecular functions (MFs), and biological processes (BPs). The Gene Ontology analysis revealed that COS reprogrammed MC3T3-E1 pre-osteoblasts to promote active bone formation by directing cellular mechanisms towards collagen-rich extracellular matrix, focal adhesion, and cytoskeletal components (cellular component level), activating molecular activities related to extracellular matrix structure, growth factors, and protease regulation (molecular function level), and engaging osteogenic transcriptional programs associated with TGF-β/BMP, Wnt, PTH/cAMP, PI3K-Akt, and MAPK pathways (at the biological process level), underscoring processes vital to osteoblast differentiation, bone matrix deposition, and mineralization (Supplementary Figure S5, Supplementary Tables S2–S4).

The enhanced CC terms indicated a spatial reorientation of the pre-osteoblast secretory system towards bone matrix formation. The extracellular region (rank 1; *p* = 8.67 × 10^−15^) and collagen-rich extracellular matrix (rank 2; *p* = 4.72 × 10^−12^) were the principal sites for TGF-β/BMP-induced collagen and ECM structural proteins, whereas the basement membrane anchored osteoblasts to the developing bone surface. The focal adhesion and cytoskeleton compartments confirmed integrin–FAK complexes that connect ECM receptor interaction to PI3K-Akt and MAPK pathways, supported by Wnt-mediated cytoskeletal rearrangement during matrix deposition.

The enriched MF terms indicated the molecular activities underlying bone matrix formation and osteogenic signaling. The structural composition of the extracellular matrix (rank 1; *p* = 9.58 × 10^−9^) and extracellular matrix binding (rank 2; *p* = 1.07 × 10^−6^) indicated TGF-β/BMP-induced collagen, fibronectin, and laminin, forming the bone matrix. Metalloendopeptidase inhibitors (TIMP family) modulated extracellular matrix remodeling downstream of TGF-β and MAPK signaling, whereas MAP kinase phosphatase activity attenuated MAPK pathways to facilitate terminal osteoblast mineralization. Growth factor activity incorporated the functions of BMP, IGF-1, and Wnt ligands in osteogenic signals, while calcium ion binding linked PTH/IP_3_R-mediated calcium release to mineralization capability.

The enhanced BP indicated a coordinated osteogenic program. The cell cycle and the inhibition of apoptosis facilitated the proliferation and survival of PI3K-Akt-driven osteoprogenitors. cAMP directly supports the PTH/cAMP/PKA/CREB transcriptional signaling pathway, while the positive control of protein phosphorylation links the PI3K-Akt and MAPK cascades to downstream osteogenic transcription. Ossification and osteoblast differentiation affirmed transcriptional commitment to the osteoblast lineage, affected by concurrent of Wnt/β-catenin, BMP/Smad, and PTH/CREB co-regulatory pathways.

### 2.6. KEGG Pathway Enrichment Analysis KEGG Pathway Analysis Under COS Treatment

According to the KEGG (Kyoto Encyclopedia of Genes and Genomes) analysis, the top 20 most significantly enriched pathways of differentially expressed genes (DEGs) primarily encompassed those related to autophagy, ubiquitin-mediated proteolysis, ribosome biogenesis in eukaryotes, and the spliceosome, among others (Figure 5B, Supplementary Table S5). Although predominantly featuring generic cellular-maintenance and cancer-associated pathways, these enrichments reveal COS’s broad impact on fundamental cellular processes, including protein quality control, mRNA processing, focal adhesion, mTOR signaling, and PI3K-Akt signaling, which intersect with osteogenic regulation. Critically, bone-related pathways demonstrate COS’s specific mechanistic action despite not ranking at the very top statistically. These include the TGF-β signaling pathway, Wnt signaling pathway, parathyroid hormone synthesis, secretion, and action, Hedgehog signaling, and ECM–receptor interaction, which were identified through targeted pathway analysis of biologically relevant DEGs (Figure 5A, Supplementary Table S5).

We also investigated osteogenic- and bone-related pathways based on biological significance, −log_10_ (Q value), and term candidate gene number from the enrichment histogram. Multiple pathways directly related to osteoblast differentiation, bone formation, and matrix mineralization appeared at various positions. These included the TGF-beta signaling pathway (rank 16), focal adhesion (rank 12), PI3K-Akt signaling pathway (rank 20), Hedgehog signaling pathway (rank 38), Hippo signaling pathway (rank 42), Wnt signaling pathway (rank 43), HIF-1 signaling pathway (rank 51), ECM–receptor interaction (rank 88), MAPK signaling pathway (rank 110), parathyroid hormone synthesis, secretion, and action (rank 103), and osteoclast differentiation (rank 173). These biologically critical pathways were interspersed among generic cellular processes and demonstrate COS’s targeted modulation of growth factor-driven osteoblastogenesis (TGF-β/BMP, Wnt, Hedgehog, Hippo), pro-survival and proliferative kinase signaling (PI3K-Akt, MAPK), mechanosensing and cytoskeletal organization (focal adhesion, ECM–receptor interaction), endocrine-coupled osteogenic signaling (parathyroid hormone), and hypoxia-responsive bone vascularization (HIF-1). These results provide mechanistic validation of the pro-osteogenic effects of COS on MC3T3-E1 pre-osteoblast cells despite variable statistical rankings (Supplementary Table S5).

### 2.7. Osteogenic Differentiation Gene Expression in Response to COS Treatment

To investigate the molecular mechanisms underlying the osteogenic effects of chitosan oligosaccharide (COS) on MC3T3-E1 pre-osteoblasts, transcriptome profiling was conducted following 21 days of osteogenic induction under 320 µg/mL of COS. Comparison of COS-treated cells with osteogenic induction medium controls revealed distinct transcriptional alterations across multiple signaling pathways central to bone formation. The identified pathways encompassed Wnt/β-catenin signaling, TGF-β/BMP signaling, PI3K-Akt/MAPK signaling and focal adhesion, and parathyroid hormone (PTH) synthesis, secretion, and action. Differentially expressed genes (DEGs) with a false discovery rate (FDR q-value) < 0.05 and a log_2_ fold change (log_2_FC) > 0.2 were considered statistically significant. The coordinated modulation of these pathways suggests that COS promotes osteogenic differentiation through the simultaneous engagement of complementary signaling cascades that regulate osteoprogenitor proliferation, osteoblast maturation, ECM deposition, and terminal mineralization, collectively supporting active bone formation in MC3T3-E1 pre-osteoblast cells (Table 2, Table 3, Table 4 and Table 5).

In MC3T3-E1 pre-osteoblast cells, treatment with chitosan oligosaccharides (COS) significantly increased canonical Wnt/β-catenin signaling, a key factor in osteogenic commitment and bone formation. A strong upregulation of Wnt ligands and receptors was observed, including *Wnt11* (wingless-type MMTV integration site family member 11; log_2_ FC = 11.11), *Wnt5b* (2.03), *Fzd6* (Frizzled receptor 6; 10.70), *Rspo2* (R-spondin 2; 11.30), and *Lgr6* (leucine-rich repeat-containing G-protein-coupled receptor 6; 1.42), suggesting increased ligand–receptor interaction at the cell surface. Downstream signaling components were similarly elevated, with *Axin2* (axis inhibition protein 2; 14.61) showing the most significant upregulation, alongside the transcription factor *Tcf7l2* (transcription factor 7-like 2; 11.30), the coactivator complex component *Tbl1xr1* (transducin beta-like 1 X-linked receptor 1; 12.19), and the co-regulator *Tle3* (transducin-like enhancer of split 3; 13.75), indicating enhanced β-catenin-dependent transcriptional activity that facilitated osteoblast differentiation. Calcium-dependent and non-canonical Wnt mediators, including Camk2a (calcium/calmodulin-dependent protein kinase II alpha; 13.17) and *Plcb4* (phospholipase C beta 4; 11.13), were elevated, indicating simultaneous activation of the Wnt/Ca^2+^ pathway. In contrast, negative regulators that drive β-catenin degradation, including *Apc* (APC regulator of WNT signaling pathway; −11.11), *Btrc* (beta-transducin repeat-containing E3 ubiquitin ligase; −11.22), and *Csnk1a1* (casein kinase 1α; −11.23) were downregulated, along with *Mapk8* (mitogen-activated protein kinase 8; −11.10), a component of the antagonistic non-canonical arm.

COS treatment significantly increased the expression of genes linked to the TGF-β signaling pathway, which is a crucial regulator of osteoblast differentiation and bone matrix formation. Upregulation of the ligand *Bmp4* (bone morphogenetic protein 4; log2 FC = 0.40) and the receptors *Tgfbr1* (transforming growth factor beta receptor 1; 13.32), Acvr2b (activin A receptor type 2B; 11.56), and *Acvr1* (1.77) was observed, indicating broad activation of receptor-level signaling. The R-Smad mediators *Smad9* (SMAD family member 9; 11.48) and *Smad2* (9.81), along with the common partner *Smad4* (11.81), were upregulated, indicating enhanced Smad complex formation and nuclear translocation. Downstream transcriptional regulators, such as *Crebbp* (CREB-binding protein; 11.48), *Tfdp1* (transcription factor *Dp-1*; 12.39), *Id1* (inhibitor of DNA binding 1; 9.25), and *Rbl1* (RB transcriptional corepressor like 1; 12.11), were similarly upregulated, indicating enhanced transcriptional activity that promotes osteoblast differentiation. Matrix-associated mediators *Dcn* (decorin; 2.25) and *Inhbb* (inhibin subunit beta B; 1.31) were also increased, as well as *Mapk3* (0.31), suggesting cross-activation of the MAPK arm of TGF-β signaling. The E3 ubiquitin ligase *Smurf1* (SMAD specific E3 ubiquitin protein ligase 1; 12.24) was increased, indicating a feedback regulatory mechanism. In contrast, the inhibitory Smads, *Smad3* (−0.73) and *Smad7* (−1.03), as well as the extracellular matrix structural protein *Fbn1* (fibrillin 1; −16.11), were downregulated. The suppression of *Smad3* and *Smad7* removes negative feedback on the Smad signaling cascade, while downregulation of *Fbn1* is proposed to release ECM-sequestered latent TGF-β/BMP ligands.

#### 2.7.1. PI3K-Akt/MAPK Signaling and Focal Adhesion Pathway

COS treatment significantly enhanced the PI3K-Akt/MAPK and focal adhesion signaling pathways in MC3T3-E1 pre-osteoblast cells, thereby facilitating cell survival, proliferation, and matrix-anchored osteogenic development. The catalytic and regulatory PI3K subunits, such as *Pik3cd* (phosphoinositide-3-kinase catalytic subunit delta; log_2_ FC = 10.58) and *Pik3cb* (10.46), and *Pik3r3* (3.81) were markedly elevated, driving downstream activation of *Pdpk1* (3-phosphoinositide-dependent protein kinase 1; 10.33), *Akt2* (AKT serine/threonine kinase 2; 6.57), and *Akt1* (1.43), indicating activation of the canonical PI3K–PDK1–Akt cascade. In parallel, the MAPK pathway component Mapk14 (mitogen-activated protein kinase 14/p38α; 1.53) was significantly upregulated, indicating concurrent activation of p38 MAPK signaling alongside the PI3K-Akt axis. Growth factor ligands and receptors associated with this pathway were similarly upregulated, including *Igf1* (insulin-like growth factor 1; 10.99), *Egf* (epidermal growth factor; 6.10), *Fgfr3* (fibroblast growth factor receptor 3; log_2_ FC = 0.72), *Osmr* (oncostatin M receptor; 10.96), *Jak1* (Janus kinase 1; 10.01), and *Vwf* (von Willebrand factor; 12.47), indicating enhanced mitogenic and pro-survival signaling upstream of the PI3K-Akt axis. Downstream effectors promoting osteogenic gene transcription and cell survival were also increased, including *Sgk1* (serum/glucocorticoid regulated kinase 1; 9.47) and *Ikbkb* (inhibitor of nuclear factor kappa B kinase subunit beta; 9.55), indicating activation of survival and inflammatory-regulatory programs that support osteoblast differentiation.

In alignment with the pathway’s integration with cell-matrix adhesion, focal adhesion kinase *Ptk2* (focal adhesion kinase 1; 12.60) exhibited significant upregulation, along with ECM structural components, such as laminins, *Lama1* (laminin subunit alpha 1; 11.34), *Lama2* (11.98), and *Lamc2* (laminin subunit gamma 2; 11.32), as well as *Col4a5* (collagen type IV alpha 5 chain; 11.52) and *Col2a1* (1.34). The matricellular protein *Spp1* (secreted phosphoprotein 1/osteopontin; 10.48) and the integrins *Itga4* (integrin subunit alpha 4; 2.95), *Itga9* (9.66), and *Itgb5* (0.76) were also elevated, indicating enhanced integrin-mediated matrix attachment that facilitated osteoblast differentiation, mechanosensing, and bone matrix production. Conversely, *Lpar1* (lysophosphatidic acid receptor 1; −9.27) and *Col9a3* (−8.97) were downregulated, indicating a decrease in pro-inflammatory signaling and promoting an optimal environment for osteogenic tissue repair.

#### 2.7.2. Parathyroid Hormone (PTH) Signaling Pathway

In MC3T3-E1 pre-osteoblast cells, COS treatment significantly influenced the parathyroid hormone (PTH) synthesis, secretion, and action pathway, suggesting activation of an endocrine-coupled osteogenic signaling pathway. Significant elevation of *Pth1r* (parathyroid hormone 1 receptor; log_2_ FC = 11.96) and the stimulatory G-protein subunit *Gnas* (GNAS complex locus/Gs-alpha; 2.41) was observed, suggesting increased receptor-level signaling coupled to intracellular cAMP production. The increased levels of downstream cAMP-dependent mediators, such as *Prkacb* (protein kinase cAMP-activated catalytic subunit beta; 12.25) and the transcription factor *Creb1* (cAMP-responsive element-binding protein 1; 10.77), suggested that the cAMP/PKA/CREB transcriptional signaling was enhanced.

The master osteogenic regulator *Runx2* (runt-related transcription factor 2; 0.74), as well as the MEF2 transcription factor family members *Mef2a* (myocyte enhancer factor 2A; 12.77), *Mef2c* (12.60), and *Mef2d* (12.19), were increased. This indicates that the transcriptional programs that facilitate osteoblast differentiation and bone formation were enhanced. The calcium release channel *Itpr1* (inositol 1,4,5-trisphosphate receptor type 1; 12.75) was significantly increased, indicating activation of calcium-dependent signaling following PTH receptor interaction. Furthermore, *Arhgef11* (Rho guanine nucleotide exchange factor 11; 11.27) and *Braf* (B-Raf proto-oncogene, serine/threonine kinase; 7.70) exhibited upregulation, indicating effects on cytoskeletal remodeling and the MAPK signaling pathway, respectively.

The matrix-remodeling enzyme *Mmp13* (matrix metallopeptidase 13; 3.36) was also increased, suggesting enhanced ECM turnover supporting bone matrix production. Additionally, upregulation of *Pde4d* (phosphodiesterase 4D; 7.18) alongside downregulation of *Pde4b* (−9.57) reflects isoform-selective regulation of cAMP degradation, in which suppression of the constitutively active *Pde4b* isoform prolongs the intracellular cAMP signal while *Pde4d* provides a delayed feedback mechanism, modulating a prolonged yet regulated PKA/CREB osteogenic transcriptional response.

## 3. Discussion

This study effectively synthesized chitosan and chitosan oligosaccharide (COS) from *Scylla olivacea* shell waste, illustrating the viability of transforming marine industry by-products into value-added biomaterials within a circular bioeconomy framework.

According to Younes and Rinaudo [8], chitin is closely linked to structural proteins, hence effective protein removal requires thorough treatment. In the deproteinization process, NaOH treatment hydrolyzes amide bonds in protein constituents, therefore diminishing the residual protein content in the resultant chitin. Prior research indicates that deproteinization with 1 M NaOH at 75 °C for roughly 16 h efficiently produces chitin with minimal protein contamination. Treatment with 4% NaOH has been demonstrated to markedly decrease protein levels, yielding a high-purity chitin product [17]. Following deproteinization, the resulting chitin was subjected to deacetylation to yield chitosan. FT-IR spectroscopy was subsequently used to verify that the polymer backbone remained structurally intact throughout this multi-step process.

FT-IR spectroscopy demonstrated that the extracted chitosan maintained the characteristic functional groups of standard chitosan, including hydroxyl (–OH), amide I/II, and glycosidic bond vibrations, indicating the preservation of the polymer backbone in Figure 1B. The determined degree of deacetylation (DD), determined from the absorbance ratio of the amide I band (1648.19 cm^−1^) to the hydroxyl band (3292.74 cm^−1^), confirmed the successful synthesis of high-quality chitosan with structural features to positive control chitosan. This study shown FT-IR spectra of the synthesized chitosan samples had significant absorption peaks around 3290–3260 cm^−1^, indicative of the stretching vibrations of hydroxyl groups, in accordance with prior studies [15]. The pattern and intensity of the absorption peaks were consistent with those documented in previous studies [7,16,17], confirming that the structural integrity of the chitosan polymer was maintained after extraction and processing.

The amide II bands of standard chitosan, crab shell-derived chitosan, and COS were observed at 1564.39, 1580.20, and 1513.89 cm^−1^, respectively, differing from the commonly cited range of 1590–1610 cm^−1^. The variation in band position may reflect differences in degree of deacetylation, source material, and processing conditions [30]. The values for standard and crab shell-derived chitosan are consistent with reported ranges of 1550–1565 cm^−1^ for chitosan [31] and approximately 1580 cm^−1^ for crustacean-derived chitosan [7], respectively. The lower COS band may be associated with hydrolysis-induced changes in chain length and intermolecular hydrogen bonding [32].

The degree of deacetylation was determined from the absorbance ratio of the amide I band to the hydroxyl band, confirming the successful synthesis of high-quality chitosan with structural features comparable to the positive control chitosan. Deacetylation is an essential process in chitosan synthesis, generally accomplished by subjecting chitin to concentrated sodium hydroxide solutions at high temperatures to eliminate acetyl groups from the polymer backbone. This leads to an abundance of free amino groups, which are known to enhance solubility, electrostatic interaction with cellular membranes, and overall biological activity [11]. Standard procedures typically utilize roughly 50% NaOH at temperatures near 100 °C to generate chitosan with a high degree of deacetylation [33]. The current study used deacetylation with 12.5 M NaOH at 70 °C for 6 h, offering a direct and dependable method for synthesizing chitosan with a significant degree of deacetylation. The DD values obtained from potentiometric titration and FT-IR spectrophotometric analysis were congruent, exceeding 80%, which signifies effective deacetylation. According to accepted categories, chitosan with deacetylation degrees of 55–70% is categorized as low, 70–85% as medium, 85–95% as high, and 95–100% as ultrahigh [10]. The chitosan generated in this work was classified as exhibiting a medium to high degree of deacetylation, similar to values documented in previous research, including a DD of 81.06% achieved with 50% NaOH at 90 °C over prolonged reaction durations [33]. In addition to the findings of Kumari et al., this study demonstrates that the degrees of deacetylation of chitosan from fish, shrimp, and crab shells were 75%, 78%, and 70%, respectively [34]. The rate and quality of deacetylation is significantly affected by NaOH concentration, temperature, and reaction time. These results indicate that an optimized conventional heating method can efficiently yield chitosan with a sufficiently high degree of deacetylation under relatively simple and economical processing conditions.

The produced chitosan and chitosan oligosaccharide (COS) samples had unique surface morphological features, according to FESEM analysis (Figure 1E). At lower magnification, the isolated chitosan displayed compact, and lamellar structures, aligning with previously documented morphologies of crustacean-derived chitosan in crab and shrimp [15,35]. At high magnifications, the chitosan extracted from crab shells had a reasonably smooth surface and small porous, whereas the standard commercial chitosan presented a considerably rougher texture. Comparable microfibrillar and porous structural characteristics have been documented for chitin and chitosan derived from *P. monodon* [36]. These architectures are beneficial for biomedical applications as they enhance surface area, thereby promoting cell interaction and adhesion and biomolecular binding. The COS generated in this work demonstrated a more refined and smoother shape, presumably indicative of its reduced molecular weight and improved solubility relative to native chitosan. The degree of deacetylation may be linked to the observed variations in surface morphology because strongly deacetylated chitosan has a higher percentage of free amine groups (-NH_2_), which help create a more homogeneous and compact structural arrangement [15]. Conversely, chitosan with considerable deacetylation preserves residual acetylated units, which may lead to heightened surface roughness. The current study revealed that both chitosan and COS displayed relatively smooth surface morphologies, indicating high material quality and effective processing, aligning with prior research that reported analogous morphological traits in well-prepared, high-purity chitosan materials.

This investigation evaluated the distinct attributes of the chitosan oligosaccharide (COS), which was the bioactive extraction from crab shell. MC3T3-E1 cells were selected as they exhibit a sequential development pattern of proliferation and differentiation, leading to the formation of calcified bone tissue. These cells are commonly utilized as an in vitro model for studying osteogenesis [22,37]. Firstly, we investigated the effect of COS extract on the cell viability. The MTT experiment revealed that the COS extract promoted MC3T3-E1 cell proliferation at all tested concentrations from 5–320 µg/mL. In addition to exhibiting pre-osteoblast effects, COS extract has been found to stimulate the growth of MC3T3-E1 at a concentration range of 1–5 mg/mL [22] and mesenchymal stem cells at concentration range of 0.05–0.5 mg/mL [38]. However, in most previous studies, natural polymer chitosan has been used to enhance osteogenic differentiation and adipogenic differentiation [39,40]. In this study, COS was prepared by hydrolysis of chitin following Songkoomkrong et al. [41], and it was used to treat MC3T3-E1 cells. No cytotoxic effects were observed on cells. Similarly, other substances such as quercitrin [42] and sciadopitysin [43] have been demonstrated to stimulate pre-osteoblast cell proliferation.

Functional assays demonstrated that COS significantly promoted alkaline phosphatase activity, extracellular matrix mineralization, and transcription and translation of osteogenic differentiation, including Runx-2 and osterix protein expression, in MC3T3-E1 cells. As shown in Figure 3C, incubation of MC3T3-E1 cells with COS (40–320 µg/mL) significantly increased ALP activity, an early-stage osteogenic differentiation marker, in the MC3T3-E1 cells. ALP produces phosphate required for mineralization and, in addition, hydrolyzes substances phosphate substrates releasing Pi, and it is associated with initiation of mineralization [44]. Calcium deposits as matrix mineralization are the final step in osteoblast differentiation and play a critical role maintaining bone tissue. In the ALP assay, ALP expression was high on Day 14 and increased significantly on Day 21 in MC3T3-E1 cells treated with 320 µg/mL COS. As shown in Figure 3A, mineralization in MC3T3-E1 cells increased in the presence of COS. The production of bone entails a complicated sequence of events, including the proliferation and differentiation of osteoprogenitor cells, ultimately leading to the creation of a mineralized extracellular matrix [37,45,46]. The late stage of osteoblast development is mineralization, during which mature osteoblasts secrete and deposit a mineral matrix primarily composed of calcium phosphate in the form of hydroxyapatite. In our findings, matrix deposition commenced in the second week of osteoblast development in COS-treated cells. Alizarin Red S staining facilitated an initial assessment of osteogenesis, while staining techniques corroborated a significant increase in mineralization by cells undergoing osteoblast differentiation following COS treatment. Consequently, COS may exert direct stimulatory effects on bone formation in cultivated MC3T3-E1 osteoblast cells.

Notably, mRNA expression levels of *ALP* and *Runx-2* in these cells were influenced by COS treatment. The pattern and degree of mRNA expression varied between treated and control cells. Moreover, COS improved the osteogenic differentiation of MC3T3-E1, potentially by inducing the expression of osteogenic genes, *ALP*, and *RUNX2*, which are pivotal in the osteogenic differentiation process [22,47,48]. Furthermore, on day 21, protein expression results were noted for osterix (OSX), a downstream mediator of RUNX2 actions [48], which is regarded as a more specific indication of high osteoblast expression, as illustrated in Figure 4B. These coordinated increases in ALP activity, matrix mineralization, and RUNX2/OSX expression suggest that COS acts through defined osteogenic signaling cascades rather than a single pathway. To identify the underlying molecular mechanisms driving these phenotypic changes, transcriptome profiling was performed on COS-treated cells.

Our investigation into the osteogenic characteristics of chitosan at a molecular level yielded intriguing results. This is the first study demonstrating the effects of chitosan oligosaccharide treatment on MC3T3-E1 cells. To initiate the examination of the molecular processes driving osteogenic differentiation prompted by COS, MC3T3-E1 cells were co-cultured with COS for a duration of 21 days. Thus, on day 21, we evaluated the gene expression in differentiated cells treated with COS in comparison to those were not treated. The enrichment analysis showed the effects of COS in gene sets. Transcriptome sequencing revealed that, among 22,833 identified genes, 19,643 were differentially expressed genes (DEGs), some crucial for osteogenic differentiation, indicating the coordinated activation of numerous osteogenesis-related signaling pathways, specifically MAPK, PI3K/Akt, and TGF-β/BMP, and focal adhesion. Upregulated genes were prominent in KEGG pathways associated with signal transduction, transcriptional control, signaling molecules, and interactions, development, and regeneration (Figure 5B, Supplementary Table S5).

Incubation with COS first increased extracellular matrix (ECM)–integrin and focal adhesion signaling, evidenced by an elevation of integrins (*Itga4*, *Itga9*, *Itgb5*) (Table 4), together with increased amounts of collagen type IV (*Col4a5*) and laminin isoforms (*Lama1*, *Lama2*, *Lamc2*). Previous studies have demonstrated that integrin mediated osteogenic signaling cascades play an important role in inducing proliferation and differentiation of osteoblasts, such as PI3K, ERK, and Wnt [49,50]. Improved cell–matrix interactions are expected to enhance osteoblast adhesion and proliferation while promoting mechano-transduction signaling. Integrin-mediated adhesion has been shown to activate downstream integrin–BMP/Smad signaling pathways in bone marrow–derived mesenchymal stem cells (BMSCs), thereby enhancing osteogenic development [51]. Similarly, in a separate model, chitosan-cartilage ECM composite scaffolds were reported to increase expression of *COL1A1*, *COL2A1*, *COL10A1*, and *ACAN* in human adipose-derived stem cells (ASCs), supporting a broader role for chitosan-based materials in promoting matrix-associated differentiation programs, including chondrogenic differentiation [52].

Downstream of these adhesion signals, the PI3K-Akt and MAPK cascades were also activated. This study indicated that chitosan treatment markedly elevated Mapk14 (mitogen-activated protein kinase 14/p38α; log_2_ FC = 1.53, FDR = 2.56 × 10^−17^) (Table 4), indicating concurrent activation of p38 MAPK signaling alongside the PI3K-Akt axis. Mapk14/p38α is a crucial regulator of osteogenic differentiation that phosphorylates and activates RUNX2, thereby augmenting the transcription of osteogenic genes such as *ALP*, *OCN*, and *Col1A1*. Col1A1 serves as an early indicator of osteogenic differentiation, with elevated expression of osteogenic genes, including collagen type I and osteocalcin, observed in mesenchymal stem cells generated from human adipose tissue (AT-MSCs) during osteogenic commitment [53]. Furthermore, the increase of ATF2 has been demonstrated to facilitate osteogenic differentiation in MSCs through the transcriptional regulation of osteogenic genes, increased alkaline phosphatase (ALP) activity, elevation of *RUNX2* and *OCN* expression, and stimulation of extracellular matrix mineralization [54,55]. Activation of the p38 MAPK pathway is acknowledged as crucial for osteoblast maturation and extracellular matrix mineralization, and its increase in COS-treated cells clearly indicates a pro-osteogenic mechanism. These results align with prior research indicating that chitosan-based hydrogels augment alkaline phosphatase activity and facilitate osteogenic differentiation of osteoblasts via the activation of JNK and p38 MAPK signaling pathways [56].

In parallel with kinase activation, transcriptional programs governing osteoblast lineage commitment were also engaged. The upregulation of Wnt ligands, receptors, and downstream effectors (Table 2), together with downregulation of negative regulators such as *Apc* and *Btrc*, indicates enhanced canonical Wnt/β-catenin signaling following COS treatment, consistent with its established role in promoting osteoblastogenesis.

Alongside Wnt activation, COS treatment also enhanced an endocrine-coupled transcriptional program, the parathyroid hormone (PTH) signaling pathway, evidenced by upregulation of Pth1r and Gnas, together with downstream cAMP/PKA/CREB mediators *Prkacb* and *Creb1* (Table 5), consistent with established PTH1R–Gsα–adenylyl cyclase–PKA–CREB signaling in osteoblasts, which promotes osteoblastic gene transcription and bone formation [57,58]. Notably, *Itpr1*, encoding an ER-associated inositol 1,4,5-trisphosphate receptor, was markedly increased, indicating parallel activation of calcium-dependent signaling. While PTH receptor signaling is classically coupled to Gs-adenylyl cyclase-PKA-CREB as evidenced by Gnas upregulation in this dataset (Table 5), the elevated Itpr1 may reflect calcium signaling contributions from concurrently activated Wnt/Ca^2+^ pathway components (e.g., *Plcb4*, *Camk2a*; Table 2) rather than direct PTH-Gq/PLC coupling, which was not independently evidenced in the present dataset. The concurrent upregulation of MEF2 family members (*Mef2a*, *Mef2c*, *Mef2d*) further supports enhanced osteoblastic transcriptional activity, since MEF2 factors are established regulators of osteoblast differentiation and bone formation [59,60]. It should be noted, however, that the relationship between PTH/cAMP/PKA signaling and MEF2 activity reported in the literature is primarily repressive, acting through salt-inducible kinase (SIK)-mediated phosphorylation of class IIa HDACs (HDAC4/5), which in turn regulates MEF2 co-repressor release [59]. The precise functional interaction between the PTH/cAMP axis and MEF2 activation observed in this dataset therefore warrants further investigation, and the two pathways are here interpreted as coordinately activated components of an endocrine-coupled osteogenic transcriptional program, rather than a direct convergent regulatory circuit. Thus, these results demonstrate that COS engages a coordinated program of ECM-adhesion, kinase, and transcriptional signaling to drive osteoblast differentiation, providing molecular support for the functional effects described above.

Osteoporosis is a highly widespread bone condition among the elderly, constituting a substantial and increasing burden on worldwide healthcare systems [61]. It is marked by systemic decrease in bone mass, strength, and microarchitecture, leading to an elevated risk of fragility fractures. Therefore, effective methods to avert bone loss and expedite bone repair in affected patients are urgently required. The results of this work expand our comprehension of the molecular processes through which chitosan oligosaccharides (COSs) serve as bioactive substances that promote osteogenic development. The reduced molecular weight of COSs imparts unique biological, chemical, and physicochemical characteristics relative to native chitosan, such as enhanced solubility and bioavailability in physiological environments [11]. These qualities allow COSs to efficiently facilitate osteoblast adhesion, spreading, proliferation, and differentiation. Our findings indicate that COSs enhance bone formation by simultaneously activating osteogenic signaling pathways, underscoring their potential utility in bone regeneration and the management of bone disorders like osteoporosis. However, in vivo and clinical investigations are necessary to assess the therapeutic efficacy, appropriate administration methods, and long-term safety of COS-based therapies in the treatment of osteoporosis.

## 4. Materials and Methods

### 4.1. Chitosan Extraction and Characterization

For chitosan extraction from shell waste, shells of mud crab, *S. olivacea*, were obtained from Coastal Aquaculture Research and Development Regional Center 2, Samut Sakhon, Thailand. The shells were thoroughly cleaned, dried in an oven at 60 °C for 6 h, and homogenized using a blender into obtain small particles (<20 mesh). The processed shell material was kept frozen until further use.

Demineralization was carried out by adding 10% HCL 1:20 (*w*/*v*) of crab shells. The reaction proceeded at room temperature under agitation at 250 rpm for 6 h. Afterwards, the demineralized shells were filtrated and washed with distilled water until neutralized. The demineralized material was subsequently immersed in ethanol for 10 min and dried at 70 °C.

Deproteinization was performed by adding 1 M NaOH to the dried demineralized shells at a solid–liquid ratio of 1:20 (*w*/*v*). Reaction was carried out under agitation at 75 °C for 16 h. The resulting solid fraction was filtrated and washed repeatedly with distilled water until neutral pH was achieved. The material was then immersed in ethanol for 10 min and dried in an oven at 60 °C to obtain chitin.

Deacetylation and chitosan production was achieved by reacting chitin with 12.5 M NaOH at a solid–liquid ratio of 1:20 (*w*/*v*) under agitation at 70 °C for 6 h. Following deacetylation, the solid product was filtered and thoroughly washed with distilled water until a neutral pH was reached. The residue that obtained was chitosan, which was dried in oven at 60 °C for 4 h (flowchart in Supplementary Figure S6).

Chitosan was characterized by FT-IR spectrophotometer (Nicolet iS50, Thermo Scientific, Carlsbad, CA, USA) and compared with standard chitosan. The obtained samples were morphologically and structurally characterized using a field emission scanning electron microscope (FESEM-Carl Zeiss/AURIGA, Jena, Germany), at the analytical equipment department, CSTE at Suranaree University of Technology.

### 4.2. Determine the Degree of Deacetylation (DD%) of Chitosan

Fourier-transform infrared spectroscopy (FT-IR) was used to find the degree of deacetylation (DD) of chitosan by looking at the absorbance ratio between the amide I band (A_1655 cm^−1^_) and the hydroxyl stretching band (A_3450 cm^−1^_). Before taking spectral measurements in the range of 4000–400 cm^−1^ at a resolution of 4 cm^−1^, dried chitosan samples were finely ground, mixed with KBr, and pressed into pellets. The following equation was used to calculate the DD [15]:

DD % = 118.883 − [(40.1647 × (A_1655_/A_3450_)] (1)

where A_1655 cm^−1^_ is the absorbance of the amide I band, which is the C=O stretching of the acetyl group. A_3450 cm^−1^_ is the absorbance of the hydroxyl (O–H) stretching band presented as a baseline.

Potentiometric titration was also carried out [62]. The chitosan sample was diluted in a specified volume of dilute acid solution to protonate the amino groups. The solution was thereafter titrated incrementally with standardized NaOH under constant agitation while observing the pH using a calibrated pH meter. The titration curve was generated by documenting the pH variations during the incremental addition of NaOH, with the equivalence (inflection) point identified from the curve.

### 4.3. Preparation of Chitosan Oligosaccharide

Crude chitosan was dried overnight at 60 °C in a hot-air oven to produce dried chitosan. The dehydrated chitosan was subsequently dissolved in 1% (*v*/*v*) acetic acid to generate a chitosan solution. Chitosan oligosaccharide (COS) was synthesized via acid hydrolysis following the protocol of Songkoomkrong et al. [41] with minor changes. Chitosan powder was dissolved in 2 M HCl and agitated continuously at 70 °C for 2 h. Subsequent to hydrolysis, the reaction mixture was allowed to cool to ambient temperature, and COS was precipitated with the addition of 100% ethanol. The mixture was subsequently incubated at 4 °C for 18 h to facilitate complete precipitation. The precipitated COS was recovered via centrifugation, washed twice with 100% ethanol to eliminate residual acid and contaminants, and dried in a hot-air oven at 70 °C until a consistent weight was achieved.

### 4.4. Cell Culture

MC3T3-E1 subclone 4 pre-osteoblasts (ATCC^®^ CRL-2593), derived from murine calvarial osteoblasts, were obtained from ATCC (Manassas, VA, USA). Cells were cultured in α-MEM (α-MEM; Thermo Fisher Scientific, Carlsbad, CA, USA) supplemented with 10% heat-inactivated fetal bovine serum (FBS, Gibco, Thermo Fisher Scientific, Carlsbad, CA, USA) and 100 U/mL penicillin, and 100 streptomycin (Gibco, Thermo Fisher Scientific, Carlsbadm, CA, USA), at 37 °C in a 5% CO_2_ incubator, in a humidified 5% CO_2_, 95% air atmosphere. Chitosan oligosaccharide (COS) was dissolved in osteogenic induction medium for treatment.

### 4.5. Cell Viability Determination

MC3T3-E1 cells (ATCC^®^ CRL-2593) were seeded in 96-well plates and cultured in MEM Alpha modified with L-glutamine, and ribo- and deoxyribonucleosides (Hyclone, Logan, UT, USA). The cells were then treated for 24 h with varying concentrations of alternan or chitosan (0, 5, 10, 20, 40, 80, 160, 320 µg/mL) on days 1, 3, 5, and 7. A 3-[4,5-dimethylthiazol-2-yl]-2,5 diphenyl tetrazolium bromide (MTT) (Biobasic, CA, USA) assay was used to determine cell viability. The cells were then incubated with MTT reagent at 37 °C for 4 h, after which dimethyl sulfoxide was added (DMSO). Absorbance at 570 nm was measured using a microplate reader (Varioskan Flash Multimode Reader, Thermo Fisher Scientific, Waltham, MA, USA).

### 4.6. Mineralization Assay and Quantification of Mineral Matrix Formation

MC3T3-E1 cells (ATCC^®^ CRL-2593) were seeded in 12-well culture plate. Post-confluence was maintained in modified MEM Alpha, with L-glutamine and ribo- and deoxyribonucleosides (Hyclone, Logan, UT, USA), containing 10% FBS (Gibco, Grand Island, NY, USA) and 1% penicillin/streptomycin (P/S) for 3 days. The cells were cultured with osteogenic differentiation medium (OstDiff medium) supplemented with 100 nM dexamethasone, 10 mM *β*-glycerophosphate, 50 mg/mL ascorbic 2-phosphate (all from Sigma-Aldrich, Louis, MO, USA), at various conditions, at chitosan concentrations of 40, 80, 160, 320 µg/mL. The medium was replaced every 3 days. After culturing for 7, 14, and 21 days, the cells were fixed with 10% formaldehyde then stained with 40 mM Alizarin Red S (ARS; Sigma-Aldrich) at room temperature. To quantify Alizarin Red S staining, the stained cells were lysed in 10% acetic acid (Sigma-Aldrich) at room temperature for 30 min. The cell lysates were then collected as supernatant after centrifugation at 15,000 rpm for 15 min and were then quantified using plate reader at absorbance 405 nm (Varioskan Flash Multimode Reader, Thermo Fisher Scientific, Waltham, MA, USA) [63,64,65].

### 4.7. Alkaline Phosphatase Assay

Alkaline phosphatase (ALP) activity was measured using a SensoLyte^®^ pNPP ALP assay kit to assess the osteogenic differentiation potential (AnaSpec, Fremont, CA, USA). The cells were maintained in an incubator at 37 °C and 5% CO_2_ with a medium change every three days. Activity of ALP was measured on days 7, 14, and 21. Briefly, the MC3T3-E1 cells were transferred from the 96-well plate to a new well. The cells were incubated for 20 min at room temperature with lysis buffer [0.1 M glycine (Bio-Rad, Hercules, CA, USA), 1% Nonidet P-40 (USB Chemical, Cleveland, OH, USA), 1 mM MgCl_2_ (Sigma-Aldrich, USA), and 1 mM ZnCl_2_ (EMSURE^®^, Merck KGaA, Darmstadt, Germany), pH 9.6]. The samples were centrifuged for 10 min at 12,000× *g* and 4 °C to separate cell debris. At an absorbance of 405 nm, the supernatant was collected for ALP activity assay using a microplate reader. The BCA assay was used to quantify total cellular protein (Thermo scientific, USA).

### 4.8. Protein Extraction and Western Blot Analysis

Expressions of osterix, an essential protein for osteogenic differentiation. Cells were lysed with RIPA buffer (0.05 M Tris–HCl, pH 7.4, 1% TritonX-100, 1% sodium deoxycholate, 0.1% SDS, 0.15 M NaCl) containing a protease and phosphatase inhibitor cocktail (Cell Signaling Technology, Danvers, MA, USA). After incubation for 20 min on ice, the lysates were centrifuged to remove cell debris. Protein concentration was determined using a Bradford assay (Bio-Rad Laboratories, Inc., Hercules, CA, USA). For Western blot analysis, equal amounts of protein (20 µg) from each sample diluted with 3× reducing SDS loading buffer (Cell Signaling Technology, USA) and a molecular weight marker (Abcam, Cambridge, UK) were loaded onto 12% sodium dodecyl sulfate polyacrylamide electrophoresis gel (SDS-PAGE). Electrophoresis was carried out at 120 V for 90 min. Proteins on SDS-PAGE gels were transferred to nitrocellulose membranes (0.45 µm pore-size; Bio-Rad, USA) using a Mini Trans-Blot^®^ Electrophoretic Transfer Cell (Bio-Rad, USA) at 120 V for 90 min. Following transfer, the membranes were blocked with 5% non-fat dry milk in Tris-buffered saline with 0.1% Tween^®^ 20 (TBST) for 1 h. Subsequently, the membranes were incubated with primary antibodies osterix antibody (1:2000 dilution; Affinity Biosciences, Changzhou, Jiangsu, China) and β-actin (1:5000; Proteintech Group, Inc., Rosemont, IL, USA) at 4 °C overnight and incubated with secondary antibodies, horseradish peroxidase (HRP)-conjugated goat anti-rabbit antibody (1:10,000 dilution; Proteintech Group, Inc., Rosemont, IL, USA) for 1 h at room temperature. The protein bands were detected with enhanced chemiluminescence (ECL) using Clarity™ Western ECL Substrate (Bio-Rad, USA). The signals were captured with an Amersham Imager 600 (GE Healthcare Life Sciences, Marlborough, MA, USA) and normalized to the intensity of the β-actin band.

### 4.9. Transcriptomic Analysis of Osteogenic Differentiation Gene Expression

To investigate the effects of chitosan oligosaccharide (COS) on gene expression, MC3T3-E1 cells were maintained in osteogenic differentiation medium (OstDiff medium) with 320 μg/mL of COS for 21 days. The total RNA was extracted from three independent biological replicates for each experimental group (OstDiff medium without COS, and OstDiff medium with COS supplement), with each replicate originating from a separate MC3T3-E1 culture, using the TriPure™ Isolation Reagent (Roche, Mannheim, Germany). The RNA concentration and RNA purity were assessed as described above. Equal amounts of RNA from the three biological replicates in each group were combined into a single pooled sample prior to library construction. Pooled samples were sent to BGI Genomics (BGI, New Territories, Hong Kong), where RNA integrity number (RIN) was assessed with an Agilent 2100 Bioanalyzer (Agilent, Santa Clara, CA, USA), and mRNA sequencing was carried out on the DNBseq platform, generating 150 bp of read length. The raw sequencing reads were processed with SOAPnuke software (version 2.3) to eliminate adapter contamination, low-quality bases, and unclear nucleotides. The clean reads were aligned to the mouse reference genome with HISAT2 software (version 2.0.4). Bowtie2 software (version 2.2.5) was used to align reads to the annotated gene set. Gene expression levels were quantified as FPKM (fragments per kilobase of transcript per million mapped reads) and TPM (transcripts per million) using RSEM (version 1.2.8). The functional characterization of the differentially expressed genes (DEGs) associated with osteogenic induction, with or without COS supplementation, was carried out following identification of differentially expressed genes using the DESeq2 package. Genes were classified as significantly differentially expressed when they met an adjusted *p* value (Q value) ≤ 0.05 together with an absolute log_2_ fold-change (FC) ≥ 0.2. To characterize the resulting phenotypic changes, enrichment analyses for Gene Ontology (GO; http://www.geneontology.org/, accessed on 7 March 2026) terms and KEGG (https://www.kegg.jp/, accessed on 7 March 2026) pathways were conducted on the annotated DEGs using a hypergeometric test implemented in Phyper. A Q value ≤ 0.05 was used as the threshold for statistical significance of enriched terms and pathways. All transcriptomic analyses were carried out through the Dr. Tom platform (https://biosys.bgi.com, accessed on 7 March 2026) to identify significantly enriched biological functions and pathways.

### 4.10. Statistical Analysis

Statistical analyzes and data visualizations were performed using GraphPad Prism 11.0 software (GraphPad Software, La Jolla, CA, USA). Comparisons between the different sample groups were evaluated using a one-way analysis of variance (ANOVA) with Turkey’s post hoc test. Each assay was performed in triplicate to ensure the accuracy and reproducibility of the results. Results with a *p*-value of less than 0.05 were considered statistically significant.

## 5. Conclusions

In conclusion, our study reveals that chitosan oligosaccharides increase osteogenic differentiation by improving osteoblast proliferation, matrix mineralization, and activation of critical osteogenic signaling pathways while reducing senescence-associated processes. Due to their low molecular weight and advantageous physicochemical characteristics, chitosan oligosaccharides are promising bioactive biomolecules for bone regeneration and the prevention or treatment of osteoporosis. Further in vivo and clinical studies are necessary to validate their therapeutic potential.

## Figures and Tables

**Table 1:** FT-IR bands and their corresponding wave numbers of the commercial and extracted chitosan.

Vibration Mode of Chitosan Shell	* Range of Standard Wavenumber (cm^−1^)	Standard Chitosan (cm^−1^)	Crab Shell Chitosan (cm^−1^)	Chitosan Oligosaccharide (cm^−1^)
**OH stretching**	3350–3360	3292.50	3292.74	3332.81
**(C-H) in pyranose ring**	2870–2900	2872.11	2874.00	2883.53
**Amide I (C=O)**	1620–1660	1649.12.	1648.19	1614.14
**Amide II (NH_2_)**	1590–1610	1564.39	1580.20	1513.89
**(CH2) in CH_2_OH group**	1420–1430	1419.65	1420.10	1414.17
**(CH3) in NHCOCH_3_ group**	1375–1382	1374.23	1375.39	1377.36
**C–O–C stretching**	1010–1070	1024.81	1026.72	1016.28
**Pyranose ring**	890–950	893.27	892.59	896.63

* The range of standard wavenumbers (cm^−1^) is similar to that obtained in the studies conducted previously by de Queiroz Antonino et al. [7], Kannan et al. [28], and Sitohy et al. [17].

**Table 2:** The differential expression of genes related to Wnt signaling pathway and MAPK pathway.

Expression	Pathway	Gene Name	Gene Description	Accession Number	Log_2_ FC	*p* Value	FDR Q Value
**Upregulation**	Wnt signaling pathway	*Axin2*	Axin 2	XM_006532060	14.61	1.90 × 10^−75^	3.23 × 10^−73^
	Wnt//MAPK signaling pathway	*Tle3*	Transducin-like enhancer of split 3	NM_001083928	13.75	7.75 × 10^−42^	4.84 × 10^−40^
	Wnt signaling pathway	*Camk2a*	Calcium/calmodulin-dependent protein kinase II alpha	NM_001286809	13.17	2.47 × 10^−28^	8.38 × 10^−27^
	Wnt//MAPK signaling pathway	*Tbl1xr1*	Transducin beta-like 1 X-linked receptor 1	XM_006535579	12.19	1.57 × 10^−14^	2.03 × 10^−13^
	Wnt signaling pathway	*Tcf7l2*	Transcription factor 7-like 2	XM_017318110	11.30	3.15 × 10^−8^	2.01 × 10^−7^
	Wnt signaling pathway	*Rspo2*	R-spondin 2	NM_172815	11.30	3.15 × 10^−8^	2.02 × 10^−7^
	Wnt signaling pathway	*Plcb4*	Phospholipase C beta 4	XM_006498947	11.13	2.51 × 10^−7^	1.42 × 10^−6^
	Wnt signaling pathway	*Wnt11*	Wingless-type MMTV integration site family member 11	XM_011241733	11.11	2.51 × 10^−7^	1.42 × 10^−6^
	Wnt signaling pathway	*Fzd6*	Frizzled class receptor 6	XM_011245454	10.70	1.58 × 10^−5^	6.68 × 10^−5^
	Wnt signaling pathway	*Wnt5b*	Wingless-type MMTV integration site family member 5B	NM_009525	2.03	2.86 × 10^−18^	5.05 × 10^−17^
	Wnt signaling pathway	*Lgr6*	Leucine-rich repeat-containing G protein-coupled receptor 6	XM_011248036	1.42	9.38 × 10^−3^	2.16 × 10^−2^
**Downregulation**	Wnt//MAPK signaling pathway	*Mapk8*	Mitogen-activated protein kinase 8 (JNK1)	NM_001310454	−11.10	2.27 × 10^−7^	1.30 × 10^−6^
	Wnt signaling pathway	*Apc*	APC regulator of WNT signaling pathway	XM_006525532	−11.11	2.27 × 10^−7^	1.29 × 10^−6^
	Wnt signaling pathway	*Btrc*	Beta-transducin repeat-containing E3 ubiquitin ligase	XM_030250716	−11.22	1.13 × 10^−7^	6.75 × 10^−7^
	Wnt signaling pathway	*Csnk1a1*	Casein kinase 1 alpha 1	XM_006526386	−11.23	5.64 × 10^−8^	3.51 × 10^−7^

**Table 3:** The differential expression of genes related to TGF-β, Wnt, and MAPK signaling pathways.

Expression	Pathway	Gene Name	Gene Description	Accession Number	Log_2_ FC	*p* Value	FDR Q Value
**Upregulation**	TGF-beta//MAPK signaling pathway	*Tgfbr1*	Transforming growth factor beta receptor 1	NM_001312869	13.32	2.47 × 10^−31^	9.64 × 10^−30^
	TGF-beta signaling pathway	*Tfdp1*	Transcription factor Dp-1	NM_001358991	12.39	1.25 × 10^−16^	1.93 × 10^−15^
	TGF-beta signaling pathway	*Smurf1*	SMAD specific E3 ubiquitin protein ligase 1	XM_011241017	12.24	3.95 × 10^−15^	5.39 × 10^−14^
	TGF-beta signaling pathway	*Rbl1*	RB transcriptional corepressor like 1	XM_006499018	12.11	6.27 × 10^−14^	7.66 × 10^−13^
	TGF-beta signaling pathway	*Smad4*	SMAD family member 4	XM_011246855	11.81	3.15 × 10^−11^	2.89 × 10^−10^
	TGF-beta signaling pathway	*Acvr2b*	Activin A receptor type 2B	XM_006511926	11.56	9.96 × 10^−10^	7.79 × 10^−9^
	TGF-beta//Wnt signaling pathway	*Crebbp*	CREB binding protein	NM_001025432	11.48	3.97 × 10^−9^	2.87 × 10^−8^
	TGF-beta signaling pathway	*Smad9*	SMAD family member 9	XM_006501728	11.48	3.97 × 10^−9^	2.88 × 10^−8^
	TGF-beta signaling pathway	*Smad2*	SMAD family member 2	XM_030250351	9.81	3.98 × 10^−3^	1.01 × 10^−2^
	TGF-beta signaling pathway	*Id1*	Inhibitor of DNA binding 1	NM_001369018	9.25	1.59 × 10^−2^	3.44 × 10^−2^
	TGF-beta signaling pathway	*Dcn*	Decorin	NM_007833	2.25	3.44 × 10^−62^	4.35 × 10^−60^
	TGF-beta signaling pathway	*Acvr1*	Activin receptor type 1	NM_001110204	1.77	4.22 × 10^−3^	1.06 × 10^−2^
	TGF-beta signaling pathway	*Inhbb*	Inhibin subunit beta B	NM_008381	1.31	1.02 × 10^−14^	1.33 × 10^−13^
	TGF-beta//MAPK signaling pathway	*Bmp4*	Bone morphogenetic protein 4	NM_007554	0.40	7.22 × 10^−10^	5.76 × 10^−9^
	TGF-beta//MAPK signaling pathway	*Mapk3*	Mitogen-activated protein kinase 3	NM_011952	0.31	2.26 × 10^−9^	1.68 × 10^−8^
**Downregulation**	TGF-beta//Wnt signaling pathway	*Smad3*	SMAD family member 3	XM_006510821	−0.73	1.33 × 10^−15^	1.89 × 10^−14^
	TGF-beta signaling pathway	*Smad7*	SMAD family member 7	NM_001042660	−1.03	3.09 × 10^−10^	2.56 × 10^−9^
	TGF-beta signaling pathway	*Fbn1*	Fibrillin 1	NM_007993	−16.11	6.36 × 10^−214^	4.83 × 10^−211^

**Table 4:** The differential expression of genes related to the PI3K-Akt/MAPK and focal adhesion signaling pathways.

Expression	Pathway	Gene Name	Gene Description	Accession Number	Log_2_ FC	*p* Value	FDR Q Value
**Upregulation**	PI3K-Akt//Focal Adhesion signaling pathway	*Ptk2*	Focal adhesion kinase 1	XM_006520430	12.60	2.49 × 10^−19^	4.71 × 10^−18^
	PI3K-Akt//Focal Adhesion signaling pathway	*Vwf*	von Willebrand factor	XM_006505918	12.47	1.57 × 10^−17^	2.62 × 10^−16^
	PI3K-Akt//Focal Adhesion signaling pathway	*Lama2*	Laminin subunit alpha 2	XM_017313815	11.98	9.94 × 10^−13^	1.08 × 10^−11^
	PI3K-Akt//Focal Adhesion signaling pathway	*Col4a5*	Collagen type IV alpha 5 chain	XM_017318372	11.52	1.99 × 10^−9^	1.50 × 10^−8^
	PI3K-Akt//Focal Adhesion signaling pathway	*Lama1*	Laminin subunit alpha 1	XM_006523717	11.34	1.58 × 10^−8^	1.05 × 10^−7^
	PI3K-Akt//Focal Adhesion signaling pathway	*Lamc2*	Laminin subunit gamma 2	XM_011247926	11.32	3.15 × 10^−8^	2.01 × 10^−7^
	PI3K-Akt//MAPK signaling pathway//Focal adhesion	*Igf1*	Insulin-like growth factor 1	XM_017313811	10.99	9.98 × 10^−7^	5.15 × 10^−6^
	PI3K-Akt signaling pathway	*Osmr*	Oncostatin M receptor	NM_001310469	10.96	1.99 × 10^−6^	9.77 × 10^−6^
	PI3K-Akt signaling pathway	*Pik3cd*	Phosphoinositide-3-kinase catalytic subunit delta	XM_006538649	10.58	3.16 × 10^−5^	1.26 × 10^−4^
	PI3K-Akt//Focal Adhesion signaling pathway	*Spp1*	Secreted phosphoprotein 1	NM_001204201	10.48	6.31 × 10^−5^	2.38 × 10^−4^
	PI3K-Akt//Focal Adhesion signaling pathway	*Pik3cb*	Phosphoinositide-3-kinase catalytic subunit beta	XM_011242829	10.46	6.31 × 10^−5^	2.37 × 10^−4^
	PI3K-Akt signaling pathway	*Pdpk1*	3-phosphoinositide-dependent protein kinase 1	XM_017317324	10.33	2.51 × 10^−4^	8.39 × 10^−4^
	PI3K-Akt signaling pathway	*Jak1*	Janus kinase 1	NM_013567	10.01	2.48 × 10^−22^	5.78 × 10^−21^
	PI3K-Akt//Focal Adhesion signaling pathway	*Itga9*	Integrin subunit alpha 9	NM_001113514	9.66	3.98 × 10^−3^	1.01 × 10^−2^
	PI3K-Akt//MAPK signaling pathway	*Ikbkb*	Inhibitor of nuclear factor kappa B kinase subunit beta	XM_030243303	9.55	7.95 × 10^−3^	1.88 × 10^−2^
	PI3K-Akt signaling pathway	*Sgk1*	Serum/glucocorticoid regulated kinase 1	NM_001161849	9.47	7.95 × 10^−3^	1.87 × 10^−2^
	PI3K-Akt//MAPK signaling pathway	*Akt2*	AKT serine/threonine kinase 2	XM_030242000	6.57	2.03 × 10^−255^	1.96 × 10^−252^
	PI3K-Akt//MAPK signaling pathway	*Egf*	Epidermal growth factor	NM_001329594	6.10	5.01 × 10^−4^	1.57 × 10^−3^
	PI3K-Akt signaling pathway	*Pik3r3*	Phosphoinositide-3-kinase regulatory subunit 3	XM_006502859	3.81	3.13 × 10^−6^	1.57 × 10^−3^
	PI3K-Akt//Focal Adhesion signaling pathway	*Itga4*	Integrin subunit alpha 4	NM_010576	2.95	2.06 × 10^−6^	1.01 × 10^−5^
	MAPK signaling pathway	*Mapk14*	Mitogen-activated protein kinase 14 (p38α)	NM_001357724	1.53	1.42 × 10^−18^	2.56 × 10^−17^
	PI3K-Akt//MAPK signaling pathway//Focal adhesion	*Akt1*	AKT serine/threonine kinase 1	XM_006515415	1.43	2.11 × 10^−7^	1.22 × 10^−6^
	PI3K-Akt//MAPK signaling pathway//Focal adhesion	*Col2a1*	Collagen type II alpha 1 chain	NM_031163	1.34	4.30 × 10^−117^	1.42 × 10^−114^
	PI3K-Akt//Focal Adhesion signaling pathway	*Itgb5*	Integrin subunit beta 5	NM_010580	0.76	1.90 × 10^−5^	7.86 × 10^−5^
	PI3K-Akt//MAPK signaling pathway	*Fgfr3*	Fibroblast growth factor receptor 3	NM_001163215	0.72	5.55 × 10^−5^	2.14 × 10^−4^
**Downregulation**	PI3K-Akt//MAPK signaling pathway	*Lpar1*	Lysophosphatidic acid receptor 1	XM_011249930	−9.27	1.54 × 10^−2^	3.38 × 10^−2^
	PI3K-Akt//MAPK signaling pathway//Focal adhesion	*Col9a3*	Collagen type IX alpha 3 chain	NM_009936	−8.97	3.08 × 10^−2^	6.22 × 10^−3^

**Table 5:** The differential expression of genes related to the parathyroid hormone (PTH) synthesis and PI3K-Akt/MAPK signaling pathways.

Expression	Pathway	Gene Name	Gene Description	Accession Number	Log_2_ FC	*p* Value	FDR Q Value
**Upregulation**	Parathyroid hormone synthesis//MAPK signaling pathway	*Mef2a*	Myocyte enhancer factor 2A	NM_001357324	12.77	9.89 × 10^−22^	2.21 × 10^−20^
	Parathyroid hormone synthesis//MAPK signaling pathway	*Itpr1*	Inositol 1,4,5-trisphosphate receptor type 1	XM_006505624	12.75	1.97 × 10^−21^	4.33 × 10^−20^
	Parathyroid hormone synthesis	*Mef2c*	Myocyte enhancer factor 2C	NM_001347577	12.60	2.49 × 10^−19^	4.71 × 10^−18^
	Parathyroid hormone synthesis	*Prkacb*	Protein kinase cAMP-activated catalytic subunit beta	NM_001164200	12.25	3.95 × 10^−15^	5.40 × 10^−14^
	Parathyroid hormone synthesis	*Mef2d*	Myocyte enhancer factor 2D	XM_011240034	12.19	1.57 × 10^−14^	2.02 × 10^−13^
	Parathyroid hormone synthesis	*Pth1r*	Parathyroid hormone 1 receptor	NM_011199	11.96	1.98 × 10^−12^	2.08 × 10^−11^
	Parathyroid hormone synthesis	*Arhgef11*	Rho guanine nucleotide exchange factor 11	XM_030252534	11.27	6.29 × 10^−8^	3.86 × 10^−7^
	Parathyroid hormone synthesis//PI3K-Akt signaling pathway	*Creb1*	cAMP-responsive element-binding protein 1	XM_011238424	10.77	7.93 × 10^−6^	3.51 × 10^−5^
	Parathyroid hormone synthesis//MAPK signaling pathway	*Braf*	B-Raf proto-oncogene, serine/threonine kinase	XM_030255095	7.70	2.51 × 10^−7^	1.41 × 10^−6^
	Parathyroid hormone synthesis	*Pde4d*	Phosphodiesterase 4D	NM_011056	7.18	5.02 × 10^−3^	1.00 × 10^−3^
	Parathyroid hormone synthesis	*Mmp13*	Matrix metallopeptidase 13	NM_008607	3.36	5.01 × 10^−4^	6.63 × 10^−5^
	Parathyroid hormone synthesis	*Gnas*	GNAS complex locus (Gs-alpha)	XM_030247568	2.41	4.07 × 10^−5^	1.59 × 10^−4^
	Parathyroid hormone synthesis	*Runx2*	Runt-related transcription factor 2	NM_001145920	0.74	7.99 × 10^−8^	1.59 × 10^−4^
**Downregulation**	Parathyroid hormone synthesis	*Pde4b*	Phosphodiesterase 4B	XM_006502856	−9.57	7.68 × 10^−3^	1.83 × 10^−2^
	Parathyroid hormone synthesis	*Gata3*	GATA binding protein 3	NM_001355112	−9.64	7.68 × 10^−3^	1.83 × 10^−2^

## Data Availability

The original contributions presented in this study are included in this article. Further inquiries can be directed to the corresponding author.

## Supplementary Materials

The following supporting information can be downloaded at https://www.mdpi.com/article/10.3390/md24090302/s1, Figure S1: Effects of COS on Osterix protein expression in preosteoblast cells during osteogenic differentiation; Figures S2–S5: Pearson correlation heatmap, Boxplot, Density plot, Gene Ontology (GO) classification; Figure S6: Flowchart depicting the sequential steps by which chitin and chitosan are extracted from crab shell; Table S1: Degree of deacetylation of chitosan standard and crab shell-derived chitosan determined by potentiometric titration and FT-IR; Tables S2–S6: DGEs identification, GO cellular function, GO molecular function, GO biological function, KEGG pathway of DEGs.

## Abbreviations

The following abbreviations are used in this manuscript:

COS	Chitosan oligosaccharide
FT-IR	Fourier-transform infrared spectroscopy
FESEM	Field emission scanning electron microscopy
DD	Degrees of deacetylation
GO	Gene Ontology
KEGG	Kyoto Encyclopedia of Genes and Genomes
DEGs	Differentially expressed genes
ATCC	American Type Culture Collection
