# Supplementary material for: Bioactive Chitosan Oligosaccharides Derived from Crab Shell Waste Stimulate Osteogenic Differentiation in Pre-Osteoblast Cells
Source: Mar Drugs. 2026 Aug 30;24(9):302. doi: 10.3390/md24090302 (PMC13608381; doi:10.3390/md24090302)
Supplement: Supplementary file 1 [file marinedrugs-24-00302-s001.zip › Supplementary Figure S1.pdf]

Supplementary Figure S1

Preosteoblast cells were treated osteogenic induction medium and COS various concentration (40,80,160,320) µg/ml, with cells maintained in MEM serving as the control. Cells were harvested on days 14 and 21 following treatment. Cells were lysed with RIPA buffer (0.05 M Tris–HCl, pH 7.4, 1% TritonX-100, 1% sodium deoxycholate, 0.1% SDS, 0.15 M NaCl) containing a protease and phosphatase inhibitor cocktail (Cell Signaling Technology, USA). After incubation for 20 min on ice, the lysates were centrifuged to remove cell debris. The protein concentration was determined following the method section and membranes were incubated overnight at 4 °C with primary antibodies osterix antibody (1:2,000 dilution; Affinity Biosciences, Jiangsu, China, and β-actin (1:5000; Proteintech Group, Inc., Rosemont, IL, USA)) and membranes were washed three times, 10 min each in TBST and probed with horseradish peroxidase (HRP)-conjugated secondary antibodies anti-rabbit at 4 °C overnight. Membranes were washed three times, 10 min each in TBST and probed with horseradish peroxidase (HRP)-conjugated goat anti-rabbit antibody (1:10,000 dilution; Proteintech Group, Inc., Rosemont, IL, USA) for 1 h at room temperature. After three TBST washes, protein bands were detected with enhanced chemiluminescence (ECL) using Clarity™ Western ECL Substrate (Bio-Rad, USA). The signals were captured with an Amersham Imager 600 (GE Healthcare Life Sciences) and normalized to the intensity of the β-actin band. All experiments were performed in three independent replicates.

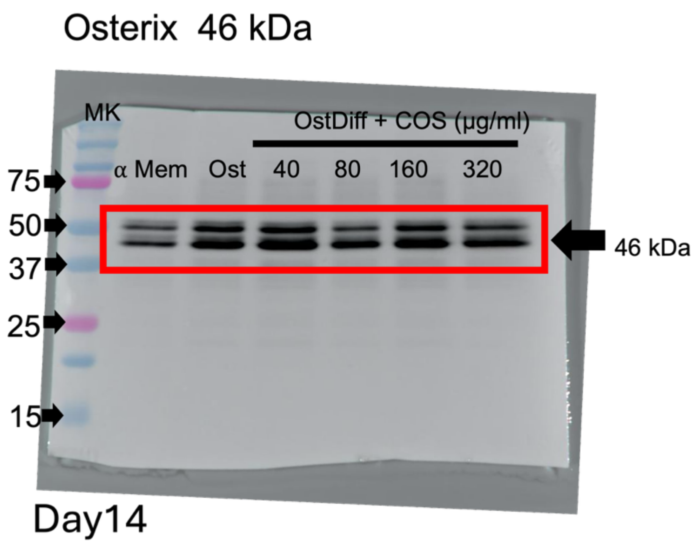

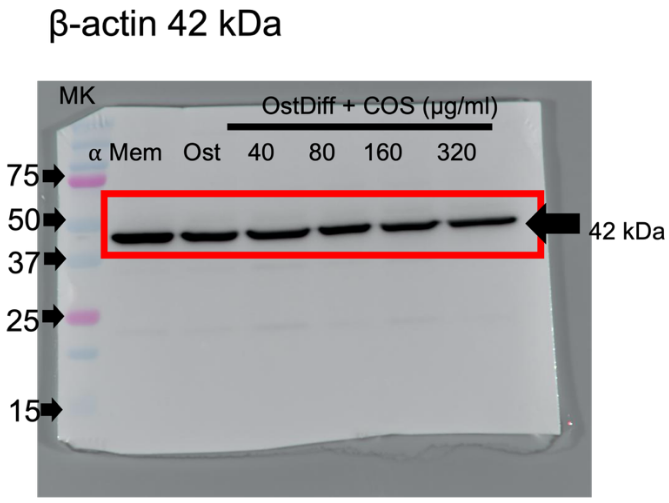

N=1; Lane 1 MK

Lane 2 Mem medium

Lane 3 Osteogenic induction medium

Lane 4 Osteogenic induction medium+COS 40 μg/ml

Lane 5 Osteogenic induction medium+COS 80 μg/ml

Lane 6 Osteogenic induction medium+COS 160 μg/ml

Lane 7 Osteogenic induction medium+COS 320 μg/ml

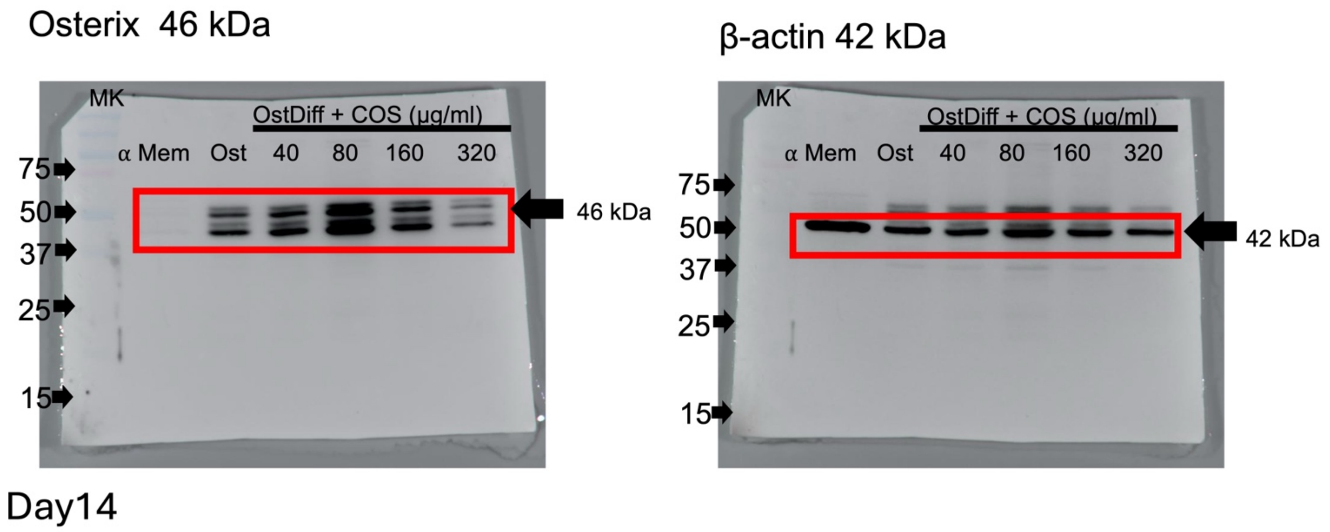

N=2; Lane 1 MK

Lane 2 Mem media

Lane 3 Osteogenic induction medium

Lane 4 Osteogenic induction medium+COS 40 μg/ml

Lane 5 Osteogenic induction medium+COS 80 μg/ml

Lane 6 Osteogenic induction medium+COS 160 μg/ml

Lane 7 Osteogenic induction medium+COS 320 μg/ml

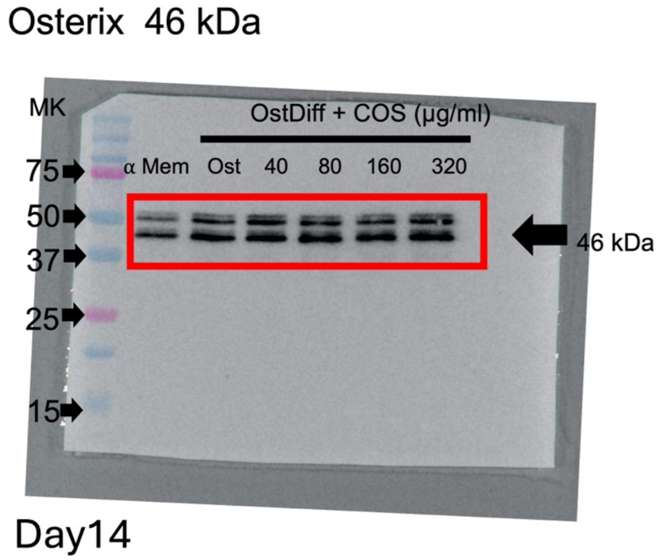

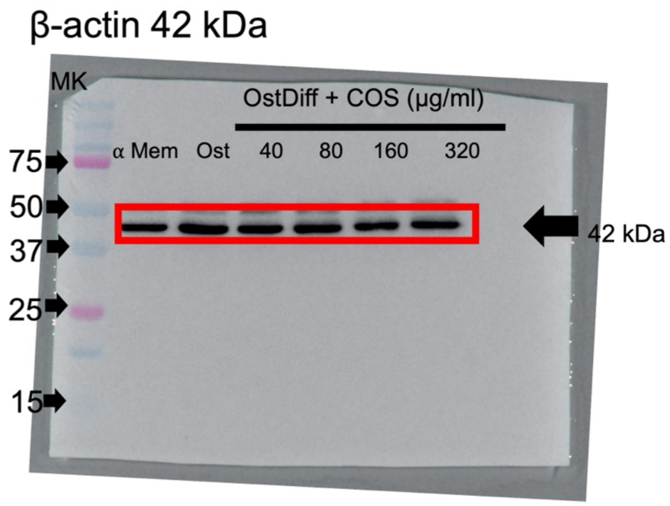

N=3; Lane 1 MK

Lane 2 Mem media

Lane 3 Osteogenic induction medium

Lane 4 Osteogenic induction medium+COS 40 μg/ml

Lane 5 Osteogenic induction medium+COS 80 μg/ml

Lane 6 Osteogenic induction medium+COS 160 μg/ml

Lane 7 Osteogenic induction medium+COS 320 μg/ml

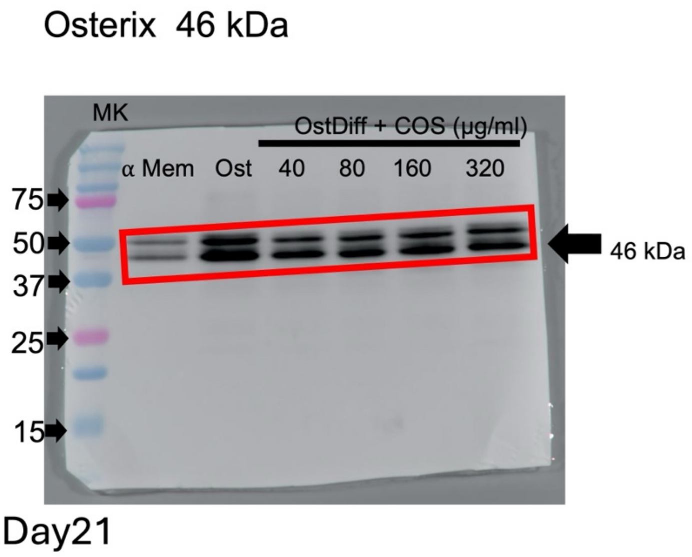

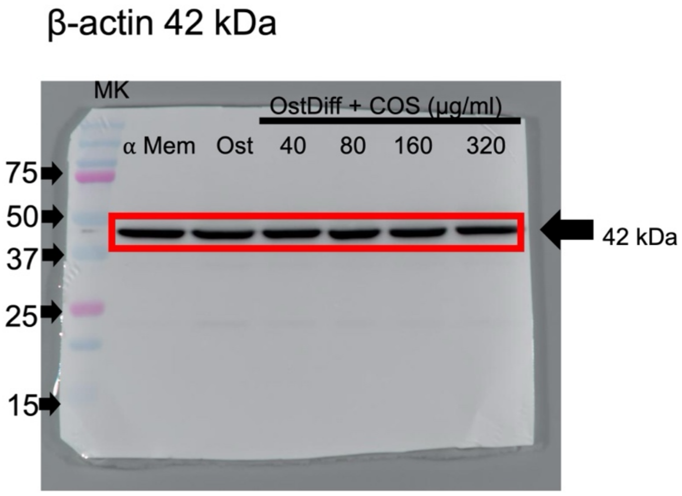

N=1; Lane 1 MK

Lane 2 Mem media

Lane 3 Osteogenic induction medium

Lane 4 Osteogenic induction medium+COS 40 μg/ml

Lane 5 Osteogenic induction medium+COS 80 μg/ml

Lane 6 Osteogenic induction medium+COS 160 μg/ml

Lane 7 Osteogenic induction medium+COS 320 μg/ml

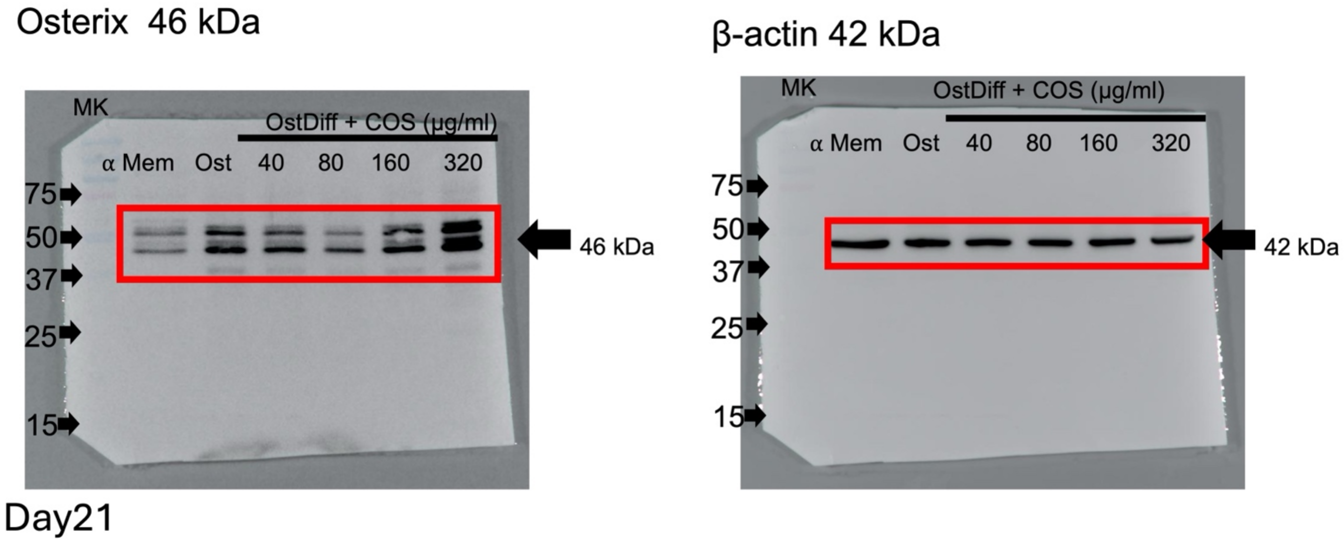

N=2; Lane 1 MK

Lane 2 Mem media

Lane 3 Osteogenic induction medium

Lane 4 Osteogenic induction medium+COS 40 µg/ml

Lane 5 Osteogenic induction medium+COS 80 µg/ml

Lane 6 Osteogenic induction medium+COS 160 µg/ml

Lane 7 Osteogenic induction medium+COS 320 µg/ml

Osterix 46 kDa

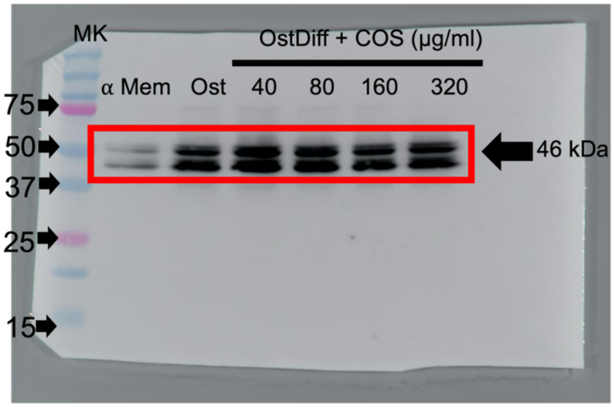

Day21

β-actin 42 kDa

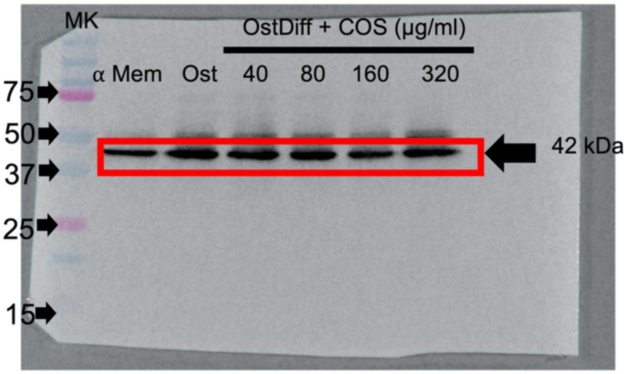

N=3; Lane 1 MK

Lane 2 Mem media

Lane 3 Osteogenic induction medium

Lane 4 Osteogenic induction medium+COS 40 μg/ml

Lane 5 Osteogenic induction medium+COS 80 μg/ml

Lane 6 Osteogenic induction medium+COS 160 μg/ml

Lane 7 Osteogenic induction medium+COS 320 μg/ml
