# Supplementary material for: Bioactive Chitosan Oligosaccharides Derived from Crab Shell Waste Stimulate Osteogenic Differentiation in Pre-Osteoblast Cells
Source: Mar Drugs. 2026 Aug 30;24(9):302. doi: 10.3390/md24090302 (PMC13608381; doi:10.3390/md24090302)
Supplement: Supplementary file 1 [file marinedrugs-24-00302-s001.zip › Supplementary Table S1.pdf]

Table Supplementary

Table S1. Degree of deacetylation (DD%) of chitosan standard and crab shell-derived chitosan determined by potentiometric titration and FT-IR

	Degree of deacetylation (DD%)	
	**Titration**	**FT-IR**
**Chitosan standard**	94.37±0.26	83.59±0.06
**crab shell chitosan**	81.65±0.69	85.78±0.37
